# Elucidating the Immune Evasion Mechanisms of *Borrelia mayonii*, the Causative Agent of Lyme Disease

**DOI:** 10.3389/fimmu.2019.02722

**Published:** 2019-11-26

**Authors:** Lea Walter, Valerie Sürth, Florian Röttgerding, Peter F. Zipfel, Karin Fritz-Wolf, Peter Kraiczy

**Affiliations:** ^1^Institute of Medical Microbiology and Infection Control, University Hospital of Frankfurt, Goethe University Frankfurt, Frankfurt, Germany; ^2^Department of Infection Biology, Leibniz-Institute for Natural Products Research and Infection Biology, Jena, Germany; ^3^Friedrich Schiller University, Jena, Germany; ^4^Max Planck Institute for Medical Research, Heidelberg, Germany; ^5^Biochemistry and Molecular Biology, Interdisciplinary Research Center, Justus Liebig University Giessen, Giessen, Germany

**Keywords:** lyme disease, spirochetes, *borrelia*, *borrelia mayonii*, innate immunity, complement, immune evasion, host cell interaction

## Abstract

*Borrelia (B.) mayonii* sp. nov. has recently been reported as a novel human pathogenic spirochete causing Lyme disease (LD) in North America. Previous data reveal a higher spirochaetemia in the blood compared to patients infected by LD spirochetes belonging to the *B. burgdorferi* sensu lato complex, suggesting that this novel genospecies must exploit strategies to overcome innate immunity, in particular complement. To elucidate the molecular mechanisms of immune evasion, we utilized various methodologies to phenotypically characterize *B. mayonii* and to identify determinants involved in the interaction with complement. Employing serum bactericidal assays, we demonstrated that *B. mayonii* resists complement-mediated killing. To further elucidate the role of the key regulators of the alternative pathway (AP), factor H (FH), and FH-like protein 1 (FHL-1) in immune evasion of *B. mayonii*, serum adsorption experiments were conducted. The data revealed that viable spirochetes recruit both regulators from human serum and FH retained its factor I-mediated C3b-inactivating activity when bound to the bacterial cells. In addition, two prominent FH-binding proteins of approximately 30 and 18 kDa were detected in *B. mayonii* strain MN14-1420. Bioinformatics identified a gene, exhibiting 60% identity at the DNA level to the *cspA* encoding gene of *B. burgdorferi*. Following PCR amplification, the gene product was produced as a His-tagged protein. The CspA-orthologous protein of *B. mayonii* interacted with FH and FHL-1, and both bound regulators promoted inactivation of C3b in the presence of factor I. Additionally, the CspA ortholog counteracted complement activation by inhibiting the alternative and terminal but not the classical and Lectin pathways, respectively. Increasing concentrations of CspA of *B. mayonii* also strongly affected C9 polymerization, terminating the formation of the membrane attack complex. To assess the role of CspA of *B. mayonii* in facilitating serum resistance, a gain-of-function strain was generated, harboring a shuttle vector allowing expression of the CspA encoding gene under its native promotor. Spirochetes producing the native protein on the cell surface overcame complement-mediated killing, indicating that CspA facilitates serum resistance of *B. mayonii*. In conclusion, here we describe the molecular mechanism utilized by *B. mayonii* to resists complement-mediated killing by capturing human immune regulators.

## Introduction

In 2016, *Borrelia (B.) mayonii* sp. nov. was identified by routine diagnostic testing of human specimens obtained from 100,595 patients in the US (1). Multi-locus sequence typing of eight housekeeping genes delineated this *Borrelia* species as a new member of the *B. burgdorferi* sensu lato (s.l.) complex (1, 2). Clinically, *B. mayonii* infected patients showed higher loads of spirochetes in the blood (10^5^-10^6^) (1) and some of them have had a focal or diffuse rash or developed neurological symptoms. So far, this species has only been identified in *Ixodes* (*I*.) *scapularis* ticks collected from the northeast and upper midwest of the US but not in *I. ricinus* from France, suggesting that *B. mayonii* is mainly distributed in the North America (2–4). Recently, experimental mice infection studies and field investigations revealed that *B. mayonii* like other Lyme disease (LD) spirochetes is maintained by transstadial transmission rather than passed by transovarial (vertical) transmission to the offspring (5). Furthermore, the probability of *B. mayonii* to be transmitted from infected *I. scapularis* ticks to the mammalian host parallels the transmission of spirochetes belonging to the *B. burgdorferi* s.l. complex. It has been shown that probability of host infection gradually increases over the duration of tick attachment, reaching 70% after 72 h of attachment and >90% after a complete tick blood meal (6–9). As potential reservoir hosts, white-footed mice (*Peromyscus leucopus*) and American red squirrel (*Tamiasciurus hudsonicus*) have been identified so far (10).

Complement as part of the innate immune system operates as a first line of defense against invading pathogenic microorganisms (11, 12). This system forms a cooperative network of more than 40 inactive precursors, membrane-bound, and fluid-phase regulators and inhibitors that discriminate between “self” from “non-self” and thereby plays a central role in the recognition and elimination of pathogenic intruders (13–17). Complement is activated by the classical (CP), the lectin (LP), and the alternative pathway (AP) (14, 16, 18, 19). Activation of the CP is primarily initiated through the interaction of C1q with IgM or IgG (13, 19). The LP is initiated by binding of mannose-binding lectin or mannan-binding lectin (MBL) or ficolins to carbohydrates on microbial cell surfaces and, in contrast to the CP and LP, the initialization of the AP is spontaneously triggered by charged moieties on microbial surfaces. Once activated, all three pathways act in concert to generate C3b, a highly reactive molecule that covalently binds to microbial surfaces by the formed C3 convertases C4b2a (CP and LP) or C3bBb (AP). Binding of C3b to the C3 convertases leads to the assembly of the C5 convertases of the CP and LP (C4b2a3b) or AP (C3bBb3b) that cleave C5. Further downstream activation is driven by linking C5b molecules to the microbial surface and a subsequent accumulation of C6, C7, C8, and C9 to create the bacteriolytic, pore-forming C5b-9 complex or membrane attack complex, MAC.

To protect self surfaces from complement activation and harmful attack of activated splice products, the complement cascade is controlled by fluid phase and surface-attached regulators or inhibitors, respectively. FH and FHL-1 are the main fluid phase regulators of the AP. Both regulators act as cofactors for factor I-mediated degradation of C3b and thereby support the dissociation (decay-accelerating activity) of the C3 convertase of the AP (18, 20). FH, a 150 kDa protein is structured into 20 complement control protein (CCP) domains of which CCP domains 1–4 are responsible for decay-accelerating and cofactor activities (20, 21). FHL-1, a 42 kDa glycoprotein, arises from alternative splicing of the *CFH* gene and is composed of the first seven CCP domains of FH (CCP 1-7) but possess four amino acids (SFTL) at the C terminus (22). Both regulators also compete with FB for binding to C3b.

Like other blood-borne pathogens, LD spirochetes have developed multiple strategies to overcome innate immunity, thereby avoiding clearance by the immune system of the respective host, e.g., by changing the surface composition or by targeting complement activation [reviewed in (23, 24)]. Two important mechanisms to combat complement activation involve the (i) recruitment of complement regulators of the AP, FH and FHL-1, to inactivate the key complement component C3b and (ii) inhibition of the assembly of the MAC by interacting with late components C7 and C9 (25–28). Concerning *B. burgdorferi*, the primary ligands involved in the acquisition of FH and FHL-1 are CspA (CRASP-1, BBA68) and CspZ (CRASP-2, BBH06), two outer surface molecules encoded by genes located on the linear plasmids lp54 and lp28-3, respectively (29–31). Both lipoproteins confer resistance to complement-mediated bacteriolysis, confirmed by studies with infectious Δ*cspA* and Δ*cspZ* knockout strains as well as non-pathogenic gain-of-function strains ectopically producing CspA or CspZ (29, 32–36). More recently it has been shown that CspA plays a role in *B. burgdorferi* survival in ticks' blood meal, resulting in tick-to-host transmission (33). This result is consistent with the fact that *cspA* is expressed in ticks and the biting site of skin (33, 37). In contrast to *cspA, cspZ* is expressed during mouse infection but the gene is downregulated in spirochetes residing in the tick midgut (33, 37). *In vivo* studies revealed that binding of FH to CspZ promotes hematogenous dissemination and tissue colonization of *B. burgdorferi* (35). Further studies also illuminated a role of CspA and CspZ in complement-driven host specificity and selective transmission which is largely based on the tailored capability of both proteins to interact with FH molecules of different animals (33, 35, 38).

Moreover, clinical examinations of positive tested specimens revealed an unusually high spirochaetemia in patients infected with *B. mayonii*, which implies that this particular borrelial species is able to survive in human blood by producing determinants to overcome complement-mediated killing. In the present study, we set out to elucidate the underlying principles of complement resistance of *B. mayonii* and to identify and functional characterize the complement interacting ligand(s).

## Materials and Methods

### Bacterial Strains, Biological and Geographical Origin, and Culture Conditions

Low-passage (<20) *B. mayonii* MN14-1420^T^ (DSMZ No. 102811, human blood, USA, deposited to culture collection by J. Petersen, CDC, USA), *B. burgdorferi* LW2 (skin, Germany), *B. burgdorferi* B31^T^ (ATCC No. 35210, tick, USA), *B. burgdorferi* Pka-1 (CSF, Germany), and *B. garinii* G1 (CSF, Germany) were cultured until mid-exponential phase (5 × 10^7^ cells per ml) at 33°C in Barbour-Stoenner-Kelly (BSK-H) medium (Bio&SELL, Feucht, Germany). Transformant G1/pCspA_Bmayo was cultured in BSK-H medium supplemented with 100 μg ml^−1^ streptomycin. *Escherichia coli* strains used as a host for propagation of the vectors and for production of recombinant hexahistidine (His_6_)-tagged proteins were grown in yeast tryptone broth at 37°C.

### Human Serum, Antibodies, and Proteins

Non-immune human serum (NHS) collected from healthy blood donors was initially tested for the presence of anti-*Borrelia* IgM and IgG antibodies as previously described (29). Only sera considered to be negative were combined to form a serum pool. All complement components were purchased from Complement Technology (Tyler, TX, USA). Polyclonal anti-FH and anti-C3 antisera were obtained from Merck Biosciences (Bad Soden, Germany) and the neoepitope-specific monoclonal anti-C5b-9 antibody was purchased from Quidel (San Diego, CA, USA). Purification of FHL-1, FH fragments containing different CCP domains, and the anti-CCP1-4 antiserum has been described previously (39). The monoclonal antibody L41 1C11 was used to detect the borrelial FlaB protein (40). Proteinase K and trypsin were purchased from Merck (Darmstadt, Germany). For the detection of His-tagged proteins, a mouse anti-His antiserum was used (GE Healthcare, Munich, Germany), and all horseradish peroxidase-conjugated immunoglobulins were purchased from Dako (Hamburg, Germany).

### Generation of Vectors and Purification of His-Tagged Proteins

The generation of vectors producing amino-terminally hexahistidine (His_6_)-tagged proteins BBA69, ErpP, and CspA of *B. burgdorferi* LW2, and BGA66 of *B. bavariensis*, respectively, are described elsewhere (29, 41–43).

The CspA-like encoding gene Bmayo_04535 (44) was amplified by PCR using primers CspA_Bmayo BamHI and CspA_Bmayo SalI (Supplementary Table 1). Following PCR amplification and digestion with appropriate restriction endonucleases, the DNA fragment was cloned into the expression vector pQE-30 Xa (Qiagen, Hilden, Germany). The resulting plasmid pQE CspA_Bmayo was then used to transform *E. coli* JM109 cells. Plasmids were sequenced to ensure that no mutations were incorporated during PCR and the cloning procedure. The production and purification of recombinant proteins have previously been described in detail (45).

To obtain a DNA fragment encompassing the CspA-like encoding gene Bmayo_04535 and the adjacent regulatory regions, genomic DNA isolated from *B. mayonii* strain MN14-1420 was used as a template for PCR with primers CspA_Bmayo_2 BamHI and CspA_Bmayo SphI (Supplementary Table 1). Following amplification and digestion, the purified DNA fragment was cloned into the shuttle vector pKFSS1 (46). Plasmids were prepared from presumptive *E. coli* clones with the Monarch plasmid kit (New England Biolabs, Frankfurt, Germany) and DNA inserts were sequenced by a commercial provider (Eurofins Genomics, Ebersberg, Germany).

### SDS-PAGE, Western Blot, and Far-Western Blot Analysis

His_6_-tagged proteins were purified by affinity chromatography, separated by 10% Tris/tricine SDS-PAGE, and transferred to nitrocellulose membranes as described previously (45). Briefly, the membranes were blocked with 5% non-fat dry milk in TBS containing 0.1% Tween 20 (TBS-T). After three wash steps with TBS-T, membranes were incubated with a monoclonal antibody L41 1C11 (anti-FlaB) (1:100) (40) or an anti-His antibody (1:3,000) followed by horseradish peroxidase-conjugated anti-mouse immunoglobulins (1:1,000). Protein-antigen complexes were detected by tetramethylbenzidine as substrate as described elsewhere (47). For the identification of FH and FHL-1 binding proteins in *Borrelia* strains and the CspA interacting domains in FH, Far-Western blotting was employed by using purified FH and FHL-1, respectively, as previously described (27). Images of the gels and nitrocellulose membranes were processed by using a GS-710 image densitometer (Bio-Rad) and the Quantity One software version 4.2.1 (Bio-Rad) (see Supplementary Figures).

### Enzyme-Linked Immunosorbent Assay (ELISA)

Nunc MaxiSorp 96-well microtiter plates (Thermo Fisher Scientific) were coated with 5 μg/ml of His_6_-tagged proteins or BSA in PBS at 4°C as described (29). Wells were washed three times with PBS containing 0.05% (v/v) Tween 20 (PBS-T) and then blocked with Blocking Buffer III BSA (AppliChem, Darmstadt, Germany). Following three washes with PBS-T, 100 μl FH or FHL-1 (5 μg/ml) were added. Following incubation for 1 h at RT, wells were washed thoroughly with PBS-T and binding of complement regulators were then assessed by incubation of the wells with a polyclonal goat anti-FH antiserum (1:1,000). After washing, protein complexes were detected by using HRP-conjugated anti-goat immunoglobulins (1:2,000). Afterwards, *o*-phenylenediamine (Merck, Darmstadt, Germany) was added to the wells and the absorbance was measured at 490 nm. Additionally, CspA of *B. mayonii* MN14-1420 was immobilized and incubated with increasing amounts of FH to determine dose-dependency of the binding and to calculate the dissociation constant.

Binding of FH to spirochetes was also assessed by ELISA. Briefly, bacterial cells (2 × 10^7^ cells) in 100 μl PBS were immobilized to Nunc MaxiSorp 96-well microtiter plates at 4°C overnight and binding of FH was detected as described above.

### C3b Degradation Assay

Factor I-mediated C3b inactivation in the presence of spirochetes was assayed after pre-incubation of 1 × 10^8^ borrelial cells with PBS supplemented with 1 μg/ml FH for 1 h at RT as described (27). Following incubation, cells were washed twice with PBS and resuspended in 30 μl PBS containing 10 μg/ml C3b and 20 μg/ml factor I. To analyze C3b degradation products by Western blotting, the bacterial cells were sedimented by centrifugation and supernatant were subjected to SDS-PAGE.

In addition, cofactor activity of FH bound to purified proteins was analyzed by monitoring factor I-mediated degradation of C3b using ELISA. Briefly, purified proteins (100 ng/ml) were immobilized on Nunc MaxiSorp 96-well microtiter plates at 4°C and after blocking, wells were incubated with FH (100 ng/ml) for 1 h at RT. After washing, PBS containing C3b (10 μg/ml) and factor I (20 μg/ml) was added. Following incubation, SDS-PAGE sample buffer was added. Each reaction was loaded on a 10% Tris/tricine SDS gel and C3b inactivation products were then analyzed by Western blotting using a polyclonal anti-C3 antibody.

### Complement Inactivation Assays

The inhibitory capacity of CspA of *B. mayonii* MN14-1420 on the classical (CP), Lectin (LP), and alternative pathway (AP) was assessed by ELISA. Nunc MaxiSorp 96-well microtiter plates were coated with either human IgM (30 ng/ml) (Merck, Darmstadt, Germany) for the CP, mannan (1 μg/ml) (Merck, Darmstadt, Germany) for the LP or LPS (100 ng/ml) (Hycult Biotech, Beutelsbach, Germany) for the AP at 4°C overnight. Following three wash steps with TBS containing 0.05% (v/v) Triton X-100 (TBS-T), wells were blocked with PBS-T for 2 h at RT. NHS (1% for the CP, 2% for the LP, and 15% for the AP) was then pre-incubated with increasing concentrations (2.5, 5, and 10 μg) of His_6_-tagged proteins for 15 min at RT before added to the wells to initiate complement activation. After washing with TBS-T, a monoclonal anti-C5b-9 antibody (1:500) (Quidel, Athens, OH; USA) was added. Following incubation for 1 h at RT, wells were washed thoroughly with TBS-T and incubated with HRP-conjugated anti-mouse immunoglobulins (1:1,000) at RT for 1 h. The reactions were developed as described above.

In order to examine the inhibitory potential of CspA of *B. mayonii* MN14-1420 on the terminal pathway, a hemolytic assay was performed as described previously (29). Briefly, sensitized sheep erythrocytes (1.5 × 10^7^ cells) were pre-incubated with C5b-6 (1.5 μg/ml) for 10 min at RT. In parallel, complement C7 (2 μg/ml), C8 (0.4 μg/ml), and C9 (2 μg/ml) were pre-incubated with or without purified His_6_-tagged proteins (2.5, 5, or 10 μg) for 5 min at RT. The pre-incubated proteins were then added to the C5b-6 coated sheep erythrocytes. Following incubation for 30 min at 37°C, erythrocytes were sedimented by centrifugation and the supernatants were transferred to a microtiter plate. Finally, the hemolysis was determined by measuring the absorbance of the supernatant at 414 nm.

### Determination of the Inhibitory Capacity of CspA of *B. mayonii* MN14-1420 on C9 Polymerization

To assess the inhibitory capacity of CspA of *B. mayonii* MN14-1420 on C9 polymerization, increasing concentrations (0.25–10 μg) of CspA of *B. mayonii* MN14-1420, CspA of *B. burgdorferi* LW2, BBA69, and BSA was incubated with C9 (3 μg) as previously described (28, 41, 48). Thereafter, auto-polymerization of C9 was induced by adding 50 μM ZnCl_2_ to each reaction mixture. As additional controls, purified C9 was incubated with or without ZnCl_2_. Reaction mixtures were then subjected to 8% Tris/Tricin-SDS gels and monomeric and polymeric C9 molecules were visualized by silver staining.

### Serum Bactericidal Assay

Spirochetes grown at mid-logarithmic phase were sedimented by centrifugation and resuspended in 500 μl BSK medium. Reaction mixtures consisting of 50 μl highly viable spirochetes (1 × 10^7^) and 50 μl of NHS were incubated at 37°C with gentle agitation. The percentage of motile and viable cells was determined by dark field microscopy after 1, 2, 4, and 6 h, respectively. Nine microscopy fields were counted for each time point per strain. Each test was performed at least three times.

### Serum Adsorption Assay

Spirochetes grown at mid-logarithmic phase were harvested and resuspended in 500 μl Gelatin veronal-buffered saline (GBS, Complement Technology, Tyler, Texas) as described previously (27). Briefly, sedimented bacteria (2 × 10^9^) were resuspended in 750 μl NHS-EDTA and incubated for 1 h at RT. After washing, the proteins bound to the bacteria surface were eluted by using 0.1 M glycine-HCl, pH 2.0. After adding 1 M Tris-HCl (pH 9.0), the cell debris were sedimented and the supernatant were analyzed by SDS-PAGE and Western blotting as previously described (27).

### Pull-Down Assay for Detecting Interacting Serum Proteins

Purified His_6_-tagged proteins were immobilized onto magnetic beads (Dynabeads^®^) as described previously (49). Briefly, recombinant proteins (40 μg) bound to magnetic particles were incubated with 500 μl NHS-EDTA for 1 h on ice and after washing, bound serum proteins were eluted with 100 mM glycine-HCl (pH 2.0). The eluate and the last wash fraction were loaded onto 10% Tris/tricine gels and proteins were detected by silver staining.

### Transformation and Characterization of Serum-Sensitive *B. garinii* Producing CspA of *B. mayonii* MN14-1420

A high-passage, non-infectious *B. garinii* strain G1 chosen as surrogate strain was grown in 100 ml BSK medium (Bio&SELL) and electrocompetent cells were prepared as described previously (30). After electroporation, spirochetes were transferred to fresh BSK medium without antibiotics (50). Following incubation for 24 h at 33°C, the cell suspension were further diluted by adding 90 ml BSK medium containing streptomycin (25 μg/ml) and 200 μl aliquots were seeded into 96-well cell culture plates. After 2–3 months, streptomycin-resistant clones could macroscopically be detected by a color change of the medium. Selected clones were then further characterized by amplifying the CspA encoding gene using primers M31 For and M31 Rev (Supplementary Table 1) as described (49).

### *In situ* Surface Assessment of CspA of *B. mayonii* MN14-1420 on Spirochetes by a Protease Degradation Assay

Localization of CspA of *B. mayonii* MN14-1420 on the surface of viable spirochetes was assessed by an *in situ* protease degradation assay as previously described (30). Borrelial cells (2 × 10^8^) were incubated with increasing concentrations of proteinase K and trypsin (6.25–50 μg/ml), respectively, for 2 h at RT. All reactions were terminated by adding 5 μl phenylmethylsulfonyl fluoride (Merck, Darmstadt, Germany) and 71 μl of cOmplete™ protease inhibitor cocktail (Merck, Darmstadt, Germany). Following sedimentation, cells were washed twice with PBS and lysed 5 times by sonication. Borrelial lysates were then subjected to 10% Tris/tricine SDS-PAGE and respective proteins were then detected by Far Western blotting (FH binding) or by Western blotting (FlaB) as described above.

### Sequence Analysis

For sequence analysis, generation of phylogenetic trees and sequence alignments, we used CLC sequence Viewer 8.0 (QIAGEN Aarhus A/S, Denmark) and EMBOSS Needle (https://www.ebi.ac.uk).

### Statistical Analysis

“Unless stated otherwise, data represent means from at least three independent experiments, and error bars indicate SD. One-way ANOVA analysis with Bonferroni's multiple comparison post test (95% confidence interval) was employed for statistical analysis using GraphPad Prism version 7. Results were deemed statistically significant for the following *p*-values: ^***^P < 0.001 and ^****^P < 0.0001” (45).

## Results

### *B. mayonii* MN14-1420 Resists Complement-Mediated Killing in Human Serum

**Figure 1:**
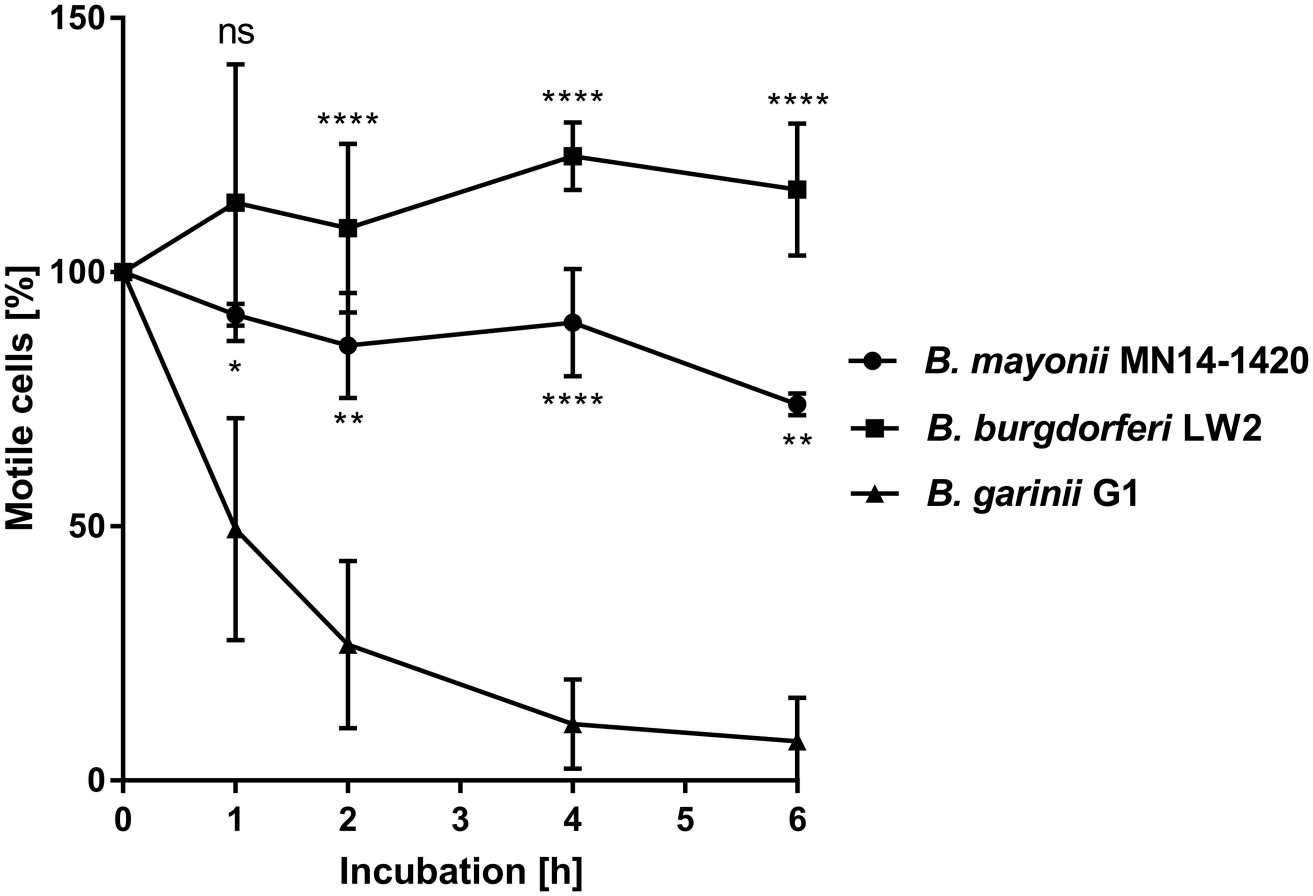
*B. mayonii* resists complement-mediated killing in human serum. Survival of *B. mayonii* MN14-1420, *B. burgdorferi* LW2, and *B. garinii* G1 in 50% NHS was monitored by dark-field microscopy. Viability and motility of borrelial cells were determined at 1, 2, 4, and 6 h. At least four independent experiments were conducted, each with very similar results. For clarity, only data from a representative experiment is shown. All experiments were performed at least four times. ^****^p ≤ 0.0001, ^**^p ≤ 0.0021, ^*^p ≤ 0.0002; one-way ANOVA with Bonferroni post test (confidence interval = 95%). ns, no statistical significance.

In contrast to other *Borrelia* species of the *B. burgdorferi* s.l. complex, *B. mayonii* causes exceptional high spirochaetemia in human blood, suggesting that this vector-borne pathogen successfully overcomes innate immunity, in particular complement. It is well-established that spirochetes belonging to the *B. burgdorferi* s.l. complex differ in their serum susceptibility pattern to human serum (51–54). To gain insight into the fundamental properties of complement evasion of this pathogen, spirochetes were treated with 50% of non-immune human serum (NHS) or heat-inactivated serum (hiNHS) and survival was assessed by counting motile cells after 1, 2, 4, and 6 h of incubation at 33°C. As shown in Figure 1, the motility and viability of the cells remained unaffected over the entire incubation period when *B. mayonii* MN14-1420 and serum-resistant strain *B. burgdorferi* LW2 were employed. As expected, more than 90% of the cells of the serum-sensitive *B. garinii* strain G1 were immotile after 6 h. In agreement with previous findings, most of these cells showed signs of fragmentations, cell lysis, and bleb formation (53). This finding suggests that *B. mayonii* MN14-1420 resists complement-mediated killing.

### *B. mayonii* MN14-1420 Interacts With Complement Regulator FH and FHL-1

**Figure 2:**
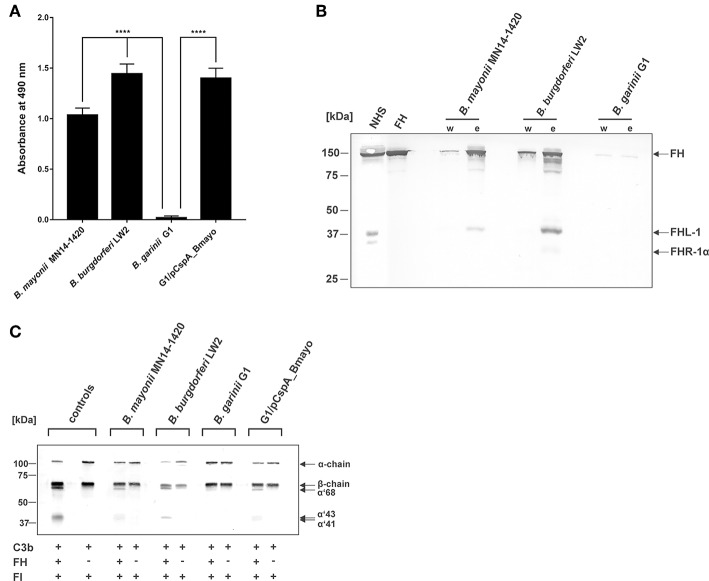
Interaction of *B. mayonii* with complement regulator FH. **(A)** Binding of purified FH to immobilized spirochetes was assessed by ELISA. Bound FH was detected using a polyclonal anti-FH antiserum (1:1,000 dilution). All experiments were performed at least four times, with each individual test carried out in triplicate. ^****^p ≤ 0.0001, one-way ANOVA with Bonferroni post test (confidence interval = 95%). **(B)** Determination of FH binding to viable spirochetes. *B. mayonii* MN14-1420, *B. burgdorferi* LW2, and *B. garinii* G1 were incubated in NHS-EDTA and cell-bound proteins were eluted. The last wash (w) and the eluate (e) fractions were separated and transferred to nitrocellulose. For detection of molecules of the FH protein family, a polyclonal anti-FH antibody (dilution 1:1,000) was applied. The mobilities of molecular mass standards are shown to the left of the panel. For better visualization of faint signals, the contrast has been increased as indicated in Supplementary Figure 4. **(C)** Determination of the regulatory activity of cell-bound FH. Factor I-mediated inactivation of C3b was assessed by the detection of C3b cleavage products. Spirochetes were incubated with purified FH and afterwards with C3b and factor I. For control purposes, all reactions were also performed in the absence of FH. The C3b fragments were visualized by Western blotting using an anti-human C3 antiserum (dilution 1:1,000). As additional controls, samples containing C3b and factor I were incubated with (+) or without (-) purified FH, respectively. The mobility of the α'- and the β-chain of C3 and the cleavage products of the α'-chain the α'68,' α'46 and α'43 fragments are indicated as well as the mobilities of molecular mass standards. A full scan of the original membrane is presented in Supplementary Figure 4.

Binding of complement regulators FH and FHL-1 is a prerequisite for all serum-resistant spirochetes except *B. bavariensis* (41) to counteract complement and overcome bacteriolysis (25, 27, 55–57). To investigate recruitment of complement regulator FH by spirochetes, cells were immobilized onto microtiter plates and binding of purified FH was assessed by ELISA. *B. mayonii* MN14-1420 and *B. burgdorferi* LW2 significantly bound FH while *B. garinii* G1 did not (Figure 2A). Having demonstrated that immobilized *B. mayonii* interacts with FH, we next examined if viable spirochetes were able to bind native complement regulators from human serum under close-to-native conditions. Cells of *B. mayonii* MN14-1420*, B. burgdorferi* LW2, and *B. garinii* G1 (each 2 × 10^9^) were incubated with 750 μl NHS-EDTA and serum proteins bound to the spirochetes were eluted by adding glycine. The reaction mixtures were then subjected to SDS-PAGE following Western blotting. As depicted in Figure 2B, *B. mayonii* MN14-1420 bound FH and FHL-1 as did control strain *B. burgdorferi* LW2. Additionally, a weak signal corresponding to FH-related protein-1α (FHR-1α) was detected in the latter borrelial strain, while *B. garinii* G1 did not bind any complement regulators. To further explore the functional activity of FH bound to the bacterial surface, factor I-mediated cleavage of C3b was assayed. Spirochetes immobilized onto microtiter plates were incubated with or without FH and after washing reaction mixtures consisting of purified factor I and C3b were added. After incubation, supernatants treated with sample buffer were loaded to 10% Tris/tricine SDS gels and C3b cleavage products were detected by Western blotting. Characteristic degradation products corresponding to the 68-, 43-, 41-kDa fragment of C3b (Figure 2C) were detected in *B. mayonii* MN14-1420 and *B. burgdorferi* LW2 but not in *B. garinii* G1, indicating that FH bound to surface of serum resistant strains retained cofactor activity. In contrast, no inactivated C3b fragments could be detected in the absence of FH.

### Identification of FH-Binding Proteins in *B. mayonii* MN14-1420

**Figure 3:**
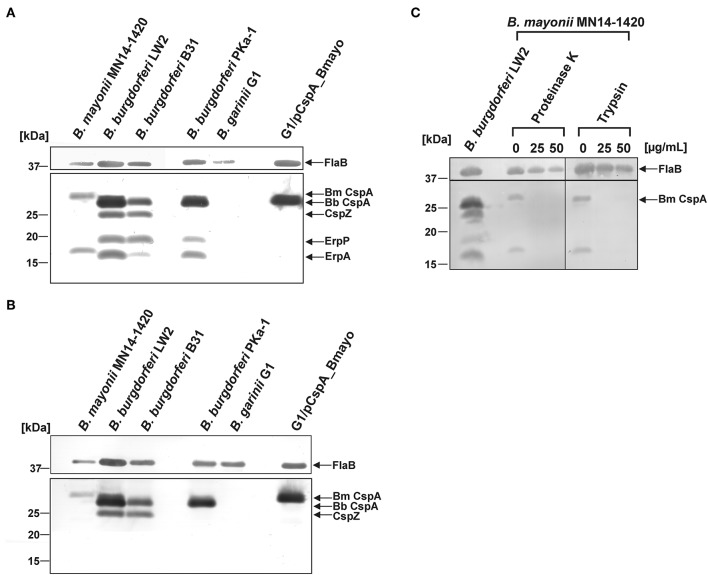
Identification and surface exposure of FH/FHL-1-binding proteins in *B. mayonii* MN14-1420. **(A,B)** Detection of FH/FHL-1-binding proteins by Far Western blot analysis using NHS as source of FH and purified FHL-1 (750 ng/ml). Cell lysates obtained from *B. mayonii* MN14-1420, *B. burgdorferi* LW2, *B. burgdorferi* B31, *B. burgdorferi* PKa-1, *B. garinii* G1, and transformant G1/pCspA_Bmayo were separated by 10% Tris/tricine-SDS-PAGE and transferred onto a nitrocellulose membrane. Flagellin (FlaB) was detected with the monoclonal antibody L41 1C11. The FH-binding proteins **(A)** were visualized by applying an anti-FH antiserum and FHL-1-binding proteins **(B)** were detected by using an anti-CCP1-4 antiserum. The corresponding to CspA protein of *B. mayonii* (Bm) MN14-1420, CspA of *B. burgdorferi* (Bb) LW2, CspZ, ErpP, and ErpA of *B. burgdorferi* s.s. are indicated at the right. **(C)** *in situ* protease accessibility assay. Native spirochetes were incubated with or without proteinase K or trypsin, then lysed by sonication and total proteins were separated by 10% Tris/tricine-SDS-PAGE. The band corresponding to CspA of *B. mayonii* is indicated on the right. The mobilities of molecular mass standards are indicated on the left. A full scan of the original membranes is presented in Supplementary Figure 5.

To identify potential FH-binding proteins in *B. mayonii* MN14-1420, Far Western blotting was employed. Two well-characterized isolates, *B. burgdorferi* B31 and *B. burgdorferi* PKa-1 (47) along with *B. burgdorferi* LW2 and *B. garinii* G1 were also included in this analysis. Cell lysates generated were separated, transferred to nitrocellulose, and incubated with NHS as a source of FH. Binding of FH was detected by a monospecific antibody. This approach identified two FH-binding proteins of approximately 30 kDa and 19 kDa produced by *B. mayonii* MN14-1420 (Figure 3A). In line with previous findings (47), four FH-binding proteins were detected in *B. burgdorferi* LW2 and *B. burgdorferi* B31 (CspA LW2, CspZ, ErpP, and ErpA) and three FH-binding proteins were identified in *B. burgdorferi* PKa-1. Of note, ErpC (18.5 kDa) is often hardly visible in *B. burgdorferi* LW2 and due to the similar molecular weight to ErpA (17.7 kDa) this particular CRASP protein is difficult to differentiate. To further characterize the FH-binding proteins, a Far Western blot was conducted using purified FHL-1 as a bait protein and the anti-CCP1-4 antiserum for detection. As depicted in Figure 3B, a single FHL-1 binding protein could be detected in cell lysates of *B. mayonii* MN14-1420 and *B. burgdorferi* PKa-1 while two FHL-1 binding proteins were found in the cell lysates of *B. burgdorferi* B31 and *B. burgdorferi* LW2. Owing to their capability to bind both regulators these CRASPs most likely correspond to CspA and CspZ.

Additionally, an *in situ* protease susceptibility assay was performed to determine localization of FH-binding proteins on the outer surface of *B. mayonii*. Viable spirochetes were incubated with 25 and 50 μg/ml of proteinase K or trypsin following preparation of cell lysates used for detection of the 30-kDa and 18-kDa protein by Far Western blotting as described above. Both proteins were highly susceptible to any protease applied while the periplasmatic FlaB protein remains unaffected during treatment, indicating that these molecules are surface exposed (Figure 3C).

Due to the similar molecular weight of the 30-kDa protein to other CspA orthologs, a nucleotide Basic Local Alignment Search Tool (BLAST) search was conducted by using the sequence of the CspA encoding gene from *B. burgdorferi* B31 (Gene ID: 1194383; protein number AAC66286.1) as a reference to find potential FH-binding proteins in *B. mayonii* MN14-1420. Two *cspA*-like genes, Bmayo_04535 and Bmayo_04540 located on linear plasmid lp54 (44) encode putative outer surface proteins of 30.2 kDa and 34.8 kDa, respectively, whereby Bmayo_04535 exhibits the highest sequence identity of 61.2% and a similarity of 75.4% to the *cspA* gene of *B. burgdorferi* B31 (Supplementary Figure 1). The Bmayo_04535 encoding gene lacking the signal sequence and the lipidation motif was PCR amplified and the DNA fragment was cloned into the pQE-30 Xa expression vector to generate a molecule carrying a hexahistidine peptide at its N-terminus. His-tagged proteins including CspA of *B. burgdorferi* LW2 as well as BBA69, and ErpP of *B. burgdorferi* B31 (29, 42, 58) as well as BGA66 of *B. bavariensis* PBi (41) were included as controls to assess differences in the complement-binding capability of this CspA-like protein. Due to the lower molecular weight (<20 kDa) and its property to bind serum-derived FH but not FHL-1, the 18-kDa protein identified (Figure 3A) most likely belong to the OspE protein family of which two (Bmayo_05450 and Bmayo_05655) encoded on cp32-1 and cp32-3 were detected by comparative genomics (44).

### CspA Orthologous Protein of *B. mayonii* MN14-1420 Interacts With Complement Regulator FH and FHL-1

**Figure 4:**
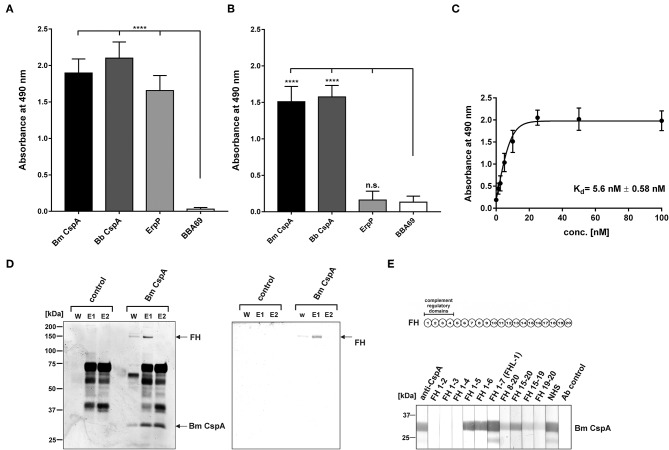
Interaction of CspA of *B. mayonii* MN14-1420 with FH and FHL-1 and mapping of the FH/FHL-1 binding site. **(A)** Binding of recombinant borrelial proteins to FH. Microtiter plates were coated with His_6_-tagged proteins, incubated with purified FH, and antigen-antibody complexes were detected using an anti-FH antiserum. All experiments were performed at least three times, with each individual test carried out in triplicate. ^****^p ≤ 0.0001, one-way ANOVA with Bonferroni post test (confidence interval = 95%). **(B)** Binding of recombinant proteins to FHL-1. Binding of purified FHL-1 was assessed by ELISA as described in **(A)**. **(C)** Dose-dependent binding of FH to CspA of *B. mayonii* MN14-1420. Recombinant CspA of *B. mayonii* MN14-1420 (5 μg/ml) was immobilized and incubated with increasing concentrations of FH. Binding curve and dissociation constant were approximated via non-linear regression, using a one-site, specific binding model. Data represent means and standard deviation of at least three different experiments, each conducted in triplicate. **(D)** FH adsorption to immobilized CspA of *B. mayonii* MN14-1420. Recombinant CspA of *B. mayonii* MN14-1420 immobilized onto magnetic particles was incubated with NHS. Empty beads were also incubated under the same conditions and used as a control to identify non-specific binding of serum proteins. After extensive washing, bound proteins were eluted with 100 mM glycine and eluate fraction e1 and e2 were separated by 10% Tris/tricine SDS-PAGE, following silver staining and Western blotting using a polyclonal anti-FH antibody. Mobilities of molecular mass standards are indicated to the left. For better visualization of faint signals, the background of the original silver stained gels was reduced as indicated in Supplementary Figure 8. **(E)** Mapping of the binding domain in FH. Schematic representation of the FH molecule (upper panel). The complement regulatory domains (CCP domains 1-4) are indicated. Localization of the CspA interacting domain in FH by Far Western blotting (lower panel). Purified CspA of *B. mayonii* (Bm) MN14-1420 was separated by 10% Tris/tricine SDS-PAGE, and transferred to nitrocellulose. The membrane strips were then incubated with constructs of FH containing different CCP domains FH1-2, FH1-3, FH1-4, FH1-5, FH1-6, FH1-7/FHL-1, FH8-20, FH15-20, FH15-19, FH19-20, NHS, and with the secondary Ab (Ab control). Bound proteins were visualized using polyclonal anti-FH antibody. A full scan of the original gel and membrane is presented in Supplementary Figure 6.

Assuming that the CspA-like protein of *B. mayonii* Bmayo_04535 displays binding properties similar to CspA of *B. burgdorferi*, we first investigated binding of FH and FHL-1 by ELISA. The CspA-like protein as well as CspA and ErpP of *B. burgdorferi* LW2 significantly bound FH (Figure 4A). In addition, the CspA-like protein and CspA of *B. burgdorferi* LW2 bound to FHL-1 while ErpP did not (Figure 4B). As expected, BBA69 neither interacted with FH nor FHL-1 (42). Further binding analysis revealed a strong binding affinity of the CspA-like protein of 5.6 nM ± 0.58 to FH (Figure 4C). Based on the identical binding properties and molecular weight to CspA of *B. burgdorferi* LW2, the 30 kDa protein was also termed CspA.

To further assess binding of serum-derived FH to the CspA protein of *B. mayonii* MN14-1420, a pull down assay was conducted. The purified His_6_-tagged CspA of *B. mayonii* MN14-1420 was immobilized to magnetic particles following incubation with inactivated NHS. Bound proteins eluted from the particles were separated by SDS-PAGE and detected by silver staining and Western blot analyses. As depicted in Figure 4D, immobilized CspA of *B. mayonii* MN14-1420 bound FH from human serum, confirming the experimental data obtained by ELISA.

Next we sought to gain further insight into the molecular interaction of the CspA protein of *B. mayonii* MN14-1420 with FH/FHL-1. In our previous investigation, we showed that CspA of *B. burgdorferi* interacts with short consensus repeats 5 through 7 of FH/FHL-1 and CCP19-20 of FH (31). In order to map the domains responsible for binding of the CspA protein of *B. mayonii* MN14-1420, a series of truncated FH constructs were used for Far Western blotting. The CspA-like protein bound constructs representing domains FH1-5, FH1-6, and FH1-7/FHL-1 as well as the construct comprising the C-terminal region FH15-20 (Figure 4E). Faint signals were also detected after incubation with constructs FH8-20, FH15-19, and FH 19-20. These findings suggest that CspA of *B. mayonii* MN14-1420 preferentially binds FH and FHL-1 by CCP5-7 and that interaction with both complement regulators did not influence their complement inhibitory activity. An additional interaction site appears to be located at the C-terminus of FH.

**Figure 5:**
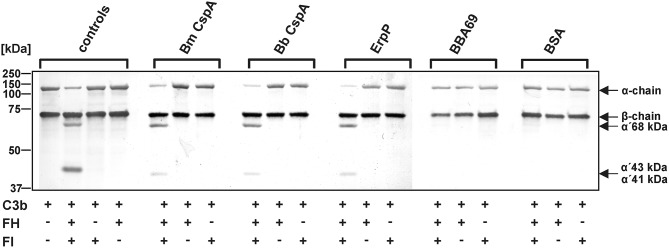
Determination of the C3b inactivation capacity of FH bound to CspA of *B. mayonii* MN14-1420. Purified proteins immobilized to microtiter plates were incubated with purified FH. After washing, C3b and factor I were added. As controls, all reactions were performed in the absence of FH or factor I (FI). All samples were subjected to Tris/tricine SDS-PAGE and transferred onto a nitrocellulose membrane. As additional controls, reaction mixtures containing different combinations of purified complement proteins were assessed. The various C3b fragments were visualized by Western blotting using an anti-human C3 antiserum (dilution 1:1,000). The mobility of the α'- and the β-chain of C3 and the cleavage products of the α'-chain the α'68,' α'46, and α'43 fragments are indicated as well as the mobilities of molecular mass standards. A full scan of the original membranes is presented in Supplementary Figure 7. For better visualization of faint signals, the contrast has been increased as indicated. Bm, *B. mayonii*; Bb, *B. burgdorferi*.

To confirm that binding of CspA of *B. mayonii* MN14-1420 to FH does not affect C3b inactivation mediated by factor I, an ELISA-based cofactor assay was conducted. Recombinant proteins coated onto microtiter plates were incubated with purified FH and after washing purified factor I and C3b were added. Each reaction mixture was then loaded to a 10% Tris/tricine SDS gels and C3b degradation products generated by FH cleavage were detected by a polyclonal antibody. The appearance of the 68-, 43-, 41-kDa fragments indicates that FH exerts cofactor activity when bound to CspA of *B. mayonii* MN14-1420, CspA of *B. burgdorferi* LW2, and ErpP (Figure 5). In contrast, none of the C3b cleavage products were detected when reactions with BBA69 and BSA were analyzed by Western blotting or in the absence of factor I.

### CspA Ortholog of *B. mayonii* MN14-1420 Inhibits Activation of the AP

**Figure 6:**
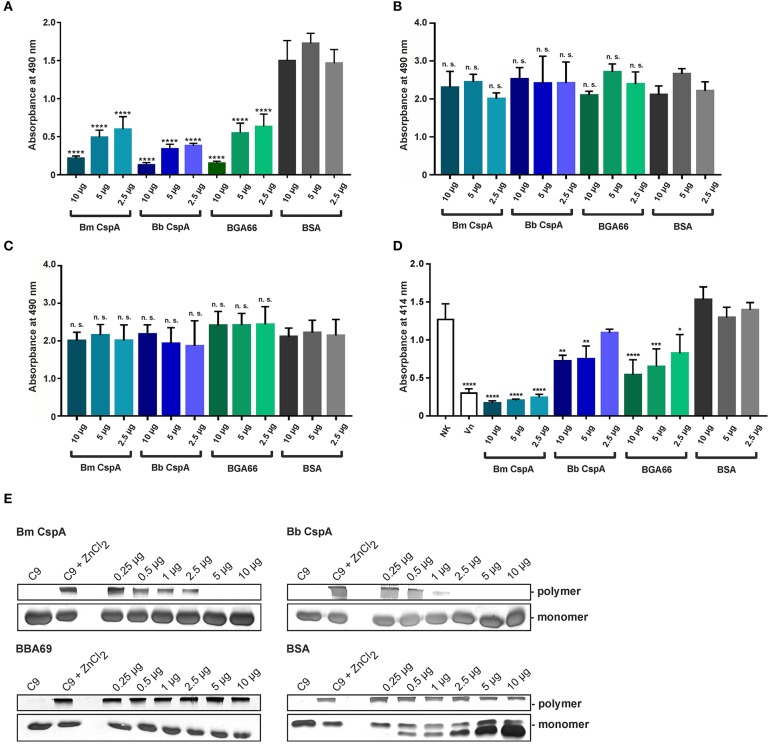
CspA of *B. mayonii* MN14-1420 terminates activation of the AP and TP and inhibits C9 polymerization. **(A–C)** Assessment of the inhibitory capacity of CspA of *B. mayonii* MN14-1420 on complement activation by an ELISA-based assay. Microtiter plates immobilized with LPS for the AP **(A)**, IgM for the CP **(B)**, and mannan for the LP **(C)** were incubated with NHS pre-incubated with increasing concentrations of recombinant proteins or BSA. After washing, formation of the MAC was detected by a monoclonal anti-C5b-9 antibody (dilution 1:500). All experiments were performed at least three times, with each individual test carried out in triplicate. ^****^p ≤ 0.0001, one-way ANOVA with Bonferroni post test (confidence interval = 95%). n.s., no statistical significance. **(D)** CspA-mediated inhibition of the TP. Sensitized sheep erythrocytes covered with the preforming C5b-6 complex were incubated with a reaction mixture containing C7, C8, and C9 that was pre-incubated with increasing concentrations of recombinant proteins or BSA. Following incubation, hemolysis of erythrocytes was detected at 414 nm. Means of three independent experiments are shown and error bars correspond to SD. Raw data were analyzed using one-way ANOVA with Bonferroni post test (confidence interval = 95%). ^****^p ≤ 0.0001; ^***^p < 0.001; ^**^p < 0.01; ^*^p < 0.05. **(E)** CspA MN14-1420 inhibits C9 polymerization. C9 was incubated with increasing concentrations of recombinant proteins or BSA and ZnCl_2_ were then added to the samples to induced polymerization. C9 incubated with and without ZnCl_2_ was used as controls. Following incubation, reactions mixtures were separated by 7.5% SDS-PAGE and C9 monomers and high molecular weight polymers were visualized by silver staining. A full scan of the original gels is presented in Supplementary Figure 8. For better visualization of faint signals in **(E)**, the contrast has been increased as indicated in Supplementary Figure 8. Bm, *B. mayonii*; Bb, *B. burgdorferi*.

To assess the inhibitory capacity of CspA of *B. mayonii* MN14-1420 on complement, the formation of the C5b-9 complex (MAC) following activation of the AP, CP, and LP by specific activators (LPS, IgM, and mannan) was detected by an ELISA-based assay. In this assay, generation of the C5b-9 complex after activation of the three pathways was measured by a neoepitope-specific antibody (59). Microtiter plates were coated with the specific activating substances and samples of NHS pre-incubated with increasing amounts of the respective proteins were added. As shown in Figure 6A, CspA of *B. mayonii* MN14-1420, CspA of *B. burgdorferi* LW2, BGA66 of *B. bavariensis* PBi inhibited the AP in a dose-dependent fashion but did not affect the CP or LP (Figures 6B,C). As expected, BSA used as control in all assays did not inhibit complement activation. These findings indicate that CspA of *B. mayonii* MN14-1420 specifically inhibits the AP.

### CspA of *B. mayonii* MN14-1420 Terminates MAC Assembly by Inhibiting Auto-Polymerization of Complement C9

Assuming that CspA of *B. mayonii* MN14-1420 might also directly interfere with MAC formation, a cell-based hemolytic assay was employed. Initially, sheep erythrocytes were incubated with the pre-forming C5b-6 complex and increasing concentrations of CspA of *B. mayonii* MN14-1420, CspA of *B. burgdorferi* LW2, BGA66 of *B. bavariensis* PBi, and BSA, respectively, followed by being incubated with complement C7, C8, and C9. Both reaction mixtures were combined and the amount of erythrocyte lysis was measured. Among the three borrelial proteins investigated, CspA of *B. mayonii* MN14-1420 most efficiently inhibited the activation of the terminal pathway (TP) and protected lysis of sheep erythrocytes from complement-mediated lysis (Figure 6D). These findings suggest that CspA of *B. mayonii* MN14-1420 mainly affects AP- and TP-activation.

Having demonstrated that CspA of *B. mayonii* MN14-1420 terminates formation of the MAC, we sought to elucidate the molecular mechanism of terminal pathway inhibition by investigating interaction of CspA of *B. mayonii* MN14-1420 to C9. Monomeric C9 is able to auto-polymerize to SDS-resistant, high molecular weight C9 polymers in the presence of divalent Zn^2+^ which can be detected by SDS-PAGE (48, 60). Initially, CspA of *B. mayonii* MN14-1420, CspA of *B. burgdorferi* LW2, BGA66 of *B. bavariensis* PBi, and BSA were allowed to bind to C9 at increasing concentrations following initiation of C9 polymerization by adding 50 μM ZnCl_2_. As an additional control, purified C9 was incubated with or without Zn^2+^ at any reaction mixture following separation through SDS-PAGE. Monomeric and polymeric C9 were then visualized by silver staining. As shown in Figure 6E, CspA of *B. mayonii* MN14-1420 and CspA of *B. burgdorferi* LW2 inhibited C9 polymerization in a dose-dependent fashion at 5.0 and 2.5 μg, respectively. By contrast, polymerization of C9 was not affected when control proteins BBA69 and BSA were assessed under identical conditions. Consistent with previous reports, C9 polymers were readily detectable after incubation with ZnCl_2_ (48, 61). This finding indicates that CspA of *B. mayonii* MN14-1420, CspA of *B. burgdorferi* LW2, and BGA66 of *B. bavariensis* PBi interfered with C9 to terminate assembly of the MAC.

### CspA of *B. mayonii* MN14-1420 Promotes Spirochete Binding to FH and Survival in Human Serum

**Figure 7:**
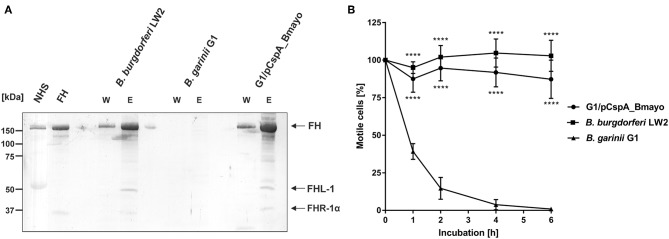
CspA of *B. mayonii* MN14-1420 facilitates resistance of *Borrelia* to complement-mediated killing. **(A)** Determination of FH binding to vital spirochetes. *B. burgdorferi* LW2, *B. garinii* G1, and *B. garinii* strain G1 producing CspA of *B. mayonii* MN14-1420 (G1/pCspA_Bmayo) were incubated in NHS-EDTA and cell-bound proteins were eluted using 0.1 M glycine. The last wash (w) and the eluate (e) fractions were separated by glycine-SDS-PAGE and transferred to nitrocellulose. For detection of molecules of the FH protein family, a polyclonal anti-FH antibody (dilution 1:1,000) was applied. The mobilities of molecular mass standards are shown to the left of the panel. A full scan of the original membranes is presented in Supplementary Figure 9. **(B)** Survival of G1/pCspA_Bmayo, *B. burgdorferi* LW2, and *B. garinii* G1 in 50% NHS was monitored by dark-field microscopy. Viability and motility of borrelial cells were determined at 1, 2, 4, and 6 h. At least four independent experiments were conducted, each with very similar results. ^****^p ≤ 0.0001; one-way ANOVA with Bonferroni post test (confidence interval = 95%).

Having demonstrated that *B. mayonii* overcome complement-mediated bacteriolysis by interacting with complement regulator FH, we sought to generate a gain-of-function *Borrelia* strain to elucidate the role of CspA of *B. mayonii* MN14-1420 independently of the 18-kDa FH-binding protein in facilitating complement resistance. Following transformation of *B. garinii* G1 with a shuttle vector carrying the CspA encoding gene with the up and downstream flanking regions, the selected transformants were assessed for ectopical production of CspA. As expected, CspA of *B. mayonii* MN14-1420 was produced on the spirochetes' surface, supported by the proteins' protease sensitivity: CspA of *B. mayonii* MN14-1420 produced by *B. garinii* G1 was highly susceptible to proteinase K at concentrations <6.25 μg/ml and nearby completely degraded 12 μg/ml trypsin K (Supplementary Figure 2). Additionally, diverse approaches revealed that transformant G1/pCspA_Bmayo efficiently bound FH (Figures 2A, 3A, 7A) and the functional assay also revealed that FH bound to the surface of G1/pCspA_Bmayo inactivates C3b in the presence of factor I (Figure 2C).

Next, serum susceptibility of CspA producing *B. garinii* cells were assessed by challenging spirochetes with NHS. We found that the majority of cells of this spirochete strains were highly motile and viable after 6 h of human serum incubation, indicating that CspA of *B. mayonii* MN14-1420 promotes spirochetes to evade complement-mediated killing in human serum (Figure 7B). By contrast, cells of the wild type *B. garinii* G1 strain were strongly affected, showing a reduction of motile cells of more than 50% after 1 h of serum challenge and nearby all cells died after 4 h. These spirochetes showed signs of cell destruction, bleb formation, and loss of motility. When using hiNHS instead of NHS, spirochetes did not show morphological changes or impact on viability by dark-field microscopy.

## Discussion

Recent studies revealed that *B. mayonii* achieves higher cell densities of spirochetes in the blood of infected patients, in contrast to the other LD causing genospecies such as *B. burgdorferi* sensu stricto (s.s.), *B. afzelii, B. garinii*, and *B. bavariensis* (1). Additionally, *B. mayonii* appears to exhibit a tropism for the central nervous system like *B. garinii* and *B. bavariensis*. These findings implicated that this novel human pathogenic genospecies developed means to successfully combat complement as the first line of defense of the human host and possess unique adhesins to bind to neuronal tissues. How *B. mayonii* resists complement-mediated bacteriolysis and what are the underlying molecular mechanisms of serum resistance are important issues that require extensive investigations. Here, we elucidated for the first time the immune evasion strategy used by *B. mayonii* to survive in human serum and identified the key molecule conferring serum resistance. The molecular mechanism of serum resistance involves inactivation of complement at the activation level of complement C3 by binding FH and FHL-1 in conjunction with the inhibition of MAC assembly by targeting C9 polymerization. These modes of action efficiently prevent opsonization and inhibit deposition of complement components on the cell surface of *B. mayonii*. Furthermore, functional analyses of a gain-of-function strain producing the key FH/FHL-1 binding protein CspA of *B. mayonii* MN14-1420 convincingly shown that this particular molecule rendered the surrogate *B. garinii* strain highly resistant to human serum.

Acquisition of fluid-phase complement regulators FH and FHL-1 are one of the prominent strategies utilized by a range of human pathogens including group A and group B streptococci as well as pneumococci (62–66), *Neisseria meningitides* (67), *N. gonorrhoeae* (68, 69), *Moraxella catarrhalis* (70), *Staphylococcus aureus* (71), *Pseudomonas aeruginosa* (72), and *Leptospira interrogans* (73) to combat the destructive effects of complement. Concerning LD spirochetes, previous investigations clearly showed that all serum-resistant *Borrelia* including *B. burgdorferi* s.s., *B. afzelii*, and *B. spielmanii* except *B. bavariensis* developed this immune evasion strategy for terminating complement activation at the most critical step of the cascade, thus allowing spirochetes to survive in the otherwise hostile environment of human blood (25, 27, 57). In this study, we demonstrate—to our knowledge for the first time—that *B. mayonii* displays a serum-resistant phenotype similar to *B. burgdorferi* s.s (Figure 1) and that resistance to complement strongly correlated with the acquisition of serum-derived immune regulators, FH and FHL-1 (Figures 2A,B, 7). More importantly, in the presence of factor I, FH bound to the borrelial surface clearly inactivates C3b (Figure 2C). The characteristic degradation products of inactivated C3b, α68-, α43-, and α41-kDa fragments were detectable upon incubation with FH but not in the absence of this complement regulator suggesting that *B. mayonii* lacks endogenous proteolytic activity to cleave C3b. This observation is in strong agreement with previous findings demonstrating that serum-resistant microorganisms degrade C3b following binding of FH and/or FHL-1 indicating that the interaction with complement regulators is of physiological relevance (25, 27, 57, 65, 71, 72).

Like other human pathogens, *Borrelia* developed additional means to overcome complement attack, e.g., by the interaction with (i) components of the CP namely C1r or C4b (74, 75), (ii) components of the TP (C7, C8, C9) (28, 41), and (iii) complement regulator C4Bp (76), respectively. Targeting different activation steps of the cascade might allow spirochetes to inhibit complement in the absence of, or with limited access to FH/FHL-1 as previously demonstrated for *B. burgdorferi* and its capacity to inhibit complement by blocking activation of the TP using FH/FHL-1 depleted human serum (28). Similar mechanisms have been described for certain parasites including *Entamoeba histolytica, Schistosoma mansoni, Trichinella spiralis*, and *Trypanosoma cruzi*, all of which are able to bind complement component of the so-called “MAC protein family” for complement inactivation (77–81). Our data strongly support the notion of a similar immune evasion strategy exploit by *B. mayonii* to terminate MAC assembly by counteracting C9 polymerization (Figure 6E).

A range of diverse FH-binding proteins have been identified so far from LD and relapsing fever borreliae including molecules exhibiting proclivities to interact with FH and FHL-1 (CspA LW2, CspZ, FhbA), FH and FHRs (ErpA, ErpC, ErpP, BsCRASP-3, Erp60, Erp61, Erp62, BhCRASP-1, HcpA, BpcA) or FH alone (CbiA) (30, 47, 49, 82–89). Among these *Borrelia*-derived proteins, CspA of *B. burgdorferi* s.s., *B. afzelii*, and *B. spielmanii*, CspZ of *B. burgdorferi* s.s., BhCRASP-1 of *B. hermsii*, BpcA of *B. parkeri*, and CbiA of *B. miyamotoi* facilitate resistance to complement-mediated killing as demonstrated by using gain-of-function or loss-of-function borrelial strains (30, 32, 33, 86, 88, 89). Using Far-Western blotting, we identified a FH/FHL-1-binding protein of approximately 30 kDa and a FH-binding protein of 19 kDa of which the 30 kDa molecule mostly resembled the CspA protein of *B. burgdorferi* and the 19 kDa protein appears to belong to the Erp protein family (Figures 3A,B). Our bioinformatic approach identified a CspA-like encoding gene located on the linear plasmid lp54 annotated as Bmayo_04535 (44) which is herein termed CspA and a gene encoding for an OspE-like protein (WP_075552645.1). The latter has a calculated molecular weight of 20.2 kDa and exhibits 77% sequence identity to the ErpA protein of *B. burgdorferi* B31. Recently, the FH binding site in ErpA and ErpC has been delineated by a computer-aided modeling of the ErpA/FH-complex based on the crystal structures of both proteins and CCP20 of FH (PDB ID codes: 3RJ3, 3SW0 and 2G7I) (90). Seven of nine residues involved in binding of OspE to FH (90) are also conserved in the identified OspE-like protein including positions Glu75, Asn79, Gly86, Thr90, Tyr120, and Asp126 suggesting that this particular molecule displays FH-binding properties (Supplementary Figure 3). Further studies will definitively clarify whether the OspE-like protein of *B. mayonii* interacts with FH and/or with other members of the FH protein family such as FHR-1, FHR-2, and FHR-5, respectively, as previously been demonstrated for ErpA, ErpC, and ErpP (49, 91).

The binding analyses showed that CspA of *B. mayonii* MN14-1420 displays a strong binding affinity of 5.6 nM to FH (Figure 4C) and confers serum resistance when ectopically produced in a serum-sensitive surrogate strain (Figure 7B). A similar FH-binding affinity of 28 nM have been reported for the CspA protein from *B. burgdorferi* s.s. (31) underscoring a preferred binding capability of these CspA orthologs to FH. Additionally, the functional characterization of the protein-protein interaction discloses the mode of action on complement. Binding of CspA of *B. mayonii* MN14-1420 via CCP domains 5 to 7 (Figure 4E) FH retains its cofactor activity for C3b inactivation (Figure 5) in the presence of factor I and also augmented the regulators' decay accelerating activity to rapidly eliminate the formed C3 convertase from the bacterial surface as also previously demonstrated for the CspA orthologous molecules of *B. burgdorferi* s.s., *B. afzelii*, and *B. spielmanii* and CspZ of *B. burgdorferi* s.s. (29, 30). The data collected with the CspA producing *B. garinii* strain confirm the findings obtained with the His-tagged protein as well as with the wild type *B. mayonii* strain as FH bound to the transformant displayed comparable regulatory activity (Figure 2C) and confers serum resistance (Figure 7B). Moreover, CspA of *B. mayonii* MN14-1420 exhibit dual inhibitory functions on complement, (i) termination of the AP by interacting with complement regulators FH/FHL-1 and (ii) blocking MAC assembly by binding to C9. As expected, CspA of *B. mayonii* MN14-1420 and CspA of *B. burgdorferi* B31 showed a similar inactivation capacity on the AP but did not target the CP and LP while CspA of *B. mayonii* MN14-1420 more strongly affected the TP compared to CspA B31 and BGA66 of *B. bavariensis* (Figure 6D). Of note, polymerization of C9 is more efficiently inhibited by CspA of *B. mayonii* MN14-1420 compared to CspA of *B. burgdorferi* LW2, thus it is tempting to speculate that CspA of *B. mayonii* block activation of the TP by interaction with additional terminal components or with pre-forming MAC complexes as previously elucidated for BGA66 and BGA71 of *B. bavariensis* PBi (41).

**Figure 8:**
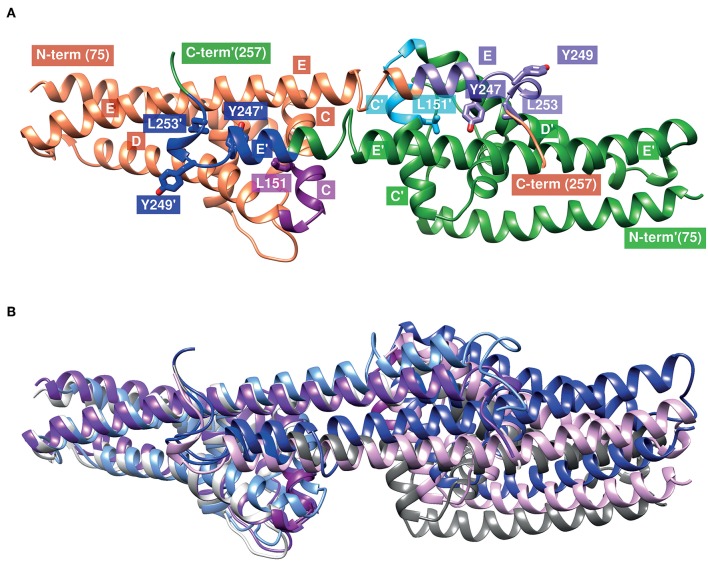
Structural model of the CspA of *B. mayonii* MN14-1420 dimer and superimposed structures of CspA of *B. burgdorferi*. **(A)** The two subunits are represented as orange or green ribbons, regions involved in binding of FHL-1 or FH are colored magenta (151–159) and violet (240–246) in the orange subunit and light blue and blue in the green subunit, respectively. Helices are marked by a capital letter, mutated residues are shown in sticks, and an apostrophe denotes the second subunit. Molecular graphics images are produced using the UCSF Chimera (92). **(B)** Superimposed structures of the CspA homodimer of both orthologs (1w33, 4bl4, 5a2u) show the different orientations of the C-terminal helix and the second subunit. The subunits in the structures of 1w33, 4bl4, and 5a2u are colored light-and dark-gray, light- and dark-magenta and light- and dark-blue, respectively. We modeled the CspA of *B. mayonii* MN14-1420 structure according to the crystal structure of CspA of *B. burgdorferi* B31 (1w33) using the Swiss-Model automated comparative protein modeling server (93).

The CspA monomer consists of five α-helices (helix A to helix E) that are connected by short loop regions to forms a “lollipop”-like structure in which the C-terminal-located helix E protrudes outwards from the pyramidal core composed of helix A to D (Figure 8) (94). Two CspA monomers assembled together by the C-terminal helices by generating a large cleft at the dimer interface (Figure 8A) (94). The extensive buried surface of the interface build by helix C and the C-terminal helix E of both subunits monomers is typical for a biologically relevant dimer and for the interaction of complement regulators. It has previously been hypothesized that the interface involving amino acid residues 146–154 located at C-terminal end of helix C and amino acid residues 233–247 at the C-terminal helix E is most likely the binding site for the interaction with both complement regulators (94, 95). By modeling the structure of CspA of *B. mayonii* MN14-1420, the homologous regions in this CspA ortholog are represented by amino acid residues 151–159 and 240–254, respectively (Figure 8A). Using *in vitro* mutagenesis, we previously identified four amino acid residues, L146, Y240, D242, and L246 in CspA of *B. burgdorferi* (Figure 8A) which drastically influence ligand binding (96). Replacement of leucine 146 (L151 in CspA of *B. mayonii* MN14-1420) with a histidine and tyrosine 240 (Y247 in CspA of *B. mayonii* MN14-1420) with an alanine strongly affect binding of FH and FHL-1. Interestingly, the leucine residue of the first subunit (L151) and the tyrosine residue of the second subunit (Y247) point into the same hydrophobic cavity (Figure 8A), and, thus it is likely that substitutions at these sides changes the orientation of the C-terminal helix. Furthermore, the substitution L246D (L253 in CspA of *B. mayonii* MN14-1420) with its additional negative charge could interact with polar or hydrophobic residues, situated on the opposed helices D and E of the other subunit, thereby changing the orientation of the C-terminal helix as well. Obviously, the position of the C-terminal helix could control the orientation of the second subunit and, thus, the overall form of the cleft between the subunits and consequently the binding of FH and FHL-1 to CspA orthologs. In accordance with our conclusions, structural superimposition (92) of the known CspA structures [1w33 (94), 4bl4 (97), 5a2u (https://www.rcsb.org/structure/5a2u)] revealed different orientations of the C-terminal helix and the second subunit (Figure 8B). It has been shown that the replacement of an aspartic acid at position 242 by a tyrosine residue results in a reduced binding capability of FHL-1 to CspA of *B. burgdorferi* (96). The homologous residue in CspA of *B. mayonii* MN14-1420 is a tyrosine residue at position 249 indicating that this particular amino acid residue does not participate in the interaction with the complement regulators. Additionally, both tyrosine residues are exposed to the solvent making it rather unlikely that they are important for binding. The findings that homologous regions are conserved in CspA of *B. mayonii* MN14-1420 in conjunction with the binding data collected strongly suggest that the same regions are engaged in the interaction with the key complement regulators of the AP.

In conclusion, this is the first study describing an immune evasion mechanism utilized by the novel blood-borne human pathogenic LD genospecies *B. mayonii*. We identified the key FH/FHL-interacting ligand, CspA of *B. mayonii* MN14-1420 as an immune evasion molecule terminating AP and TP activation. At least two modes of action were delineated for this particular protein: (i) acquisition of FH and FHL-1 following factor I-mediated degradation of C3b and (ii) inhibition of MAC assembly by direct binding to C9. More importantly, heterologous production of CspA protects spirochetes from the deleterious effects of complement and renders serum susceptible spirochetes fully resistant. It will be interesting to determine at which time point the CspA encoding gene is expressed during the tick-to-host infection cycle and whether the expression pattern matches that of CspA of *B. burgdorferi*. To further explore the role of CspA within the infection process of *B. mayonii*, a virulent loss-of-function or a gain-of-function strain will be necessary.

## Data Availability Statement

All datasets generated for this study are included in the article/Supplementary Material.

## Ethics Statement

The studies involving human participants were reviewed and approved by Ethics committee of the University Hospital Frankfurt (control number 160/10 and 222/14), Goethe University of Frankfurt am Main. The patients/participants provided their written informed consent to participate in this study in accordance with the Declaration of Helsinki.

## Author Contributions

LW, VS, and FR: study design, experimental work, data interpretation, figure preparation, and final approval. KF-W: structure analysis, data interpretation, figure preparation, and final approval. PZ: contribution of reagents, critical reading of the manuscript, and final approval. PK: study design, data interpretation, figure and table preparation, drafting the article, wrote the manuscript, and final approval.

### Conflict of Interest

The authors declare that the research was conducted in the absence of any commercial or financial relationships that could be construed as a potential conflict of interest.
