# Supplementary material for: Elucidating the Immune Evasion Mechanisms of Borrelia mayonii, the Causative Agent of Lyme Disease
Source: Front Immunol. 2019 Nov 26;10:2722. doi: 10.3389/fimmu.2019.02722 (PMC6902028; doi:10.3389/fimmu.2019.02722)
Supplement: Supplementary file 1 [file Data_Sheet_1.PDF]

Supplementary Figure 1

A

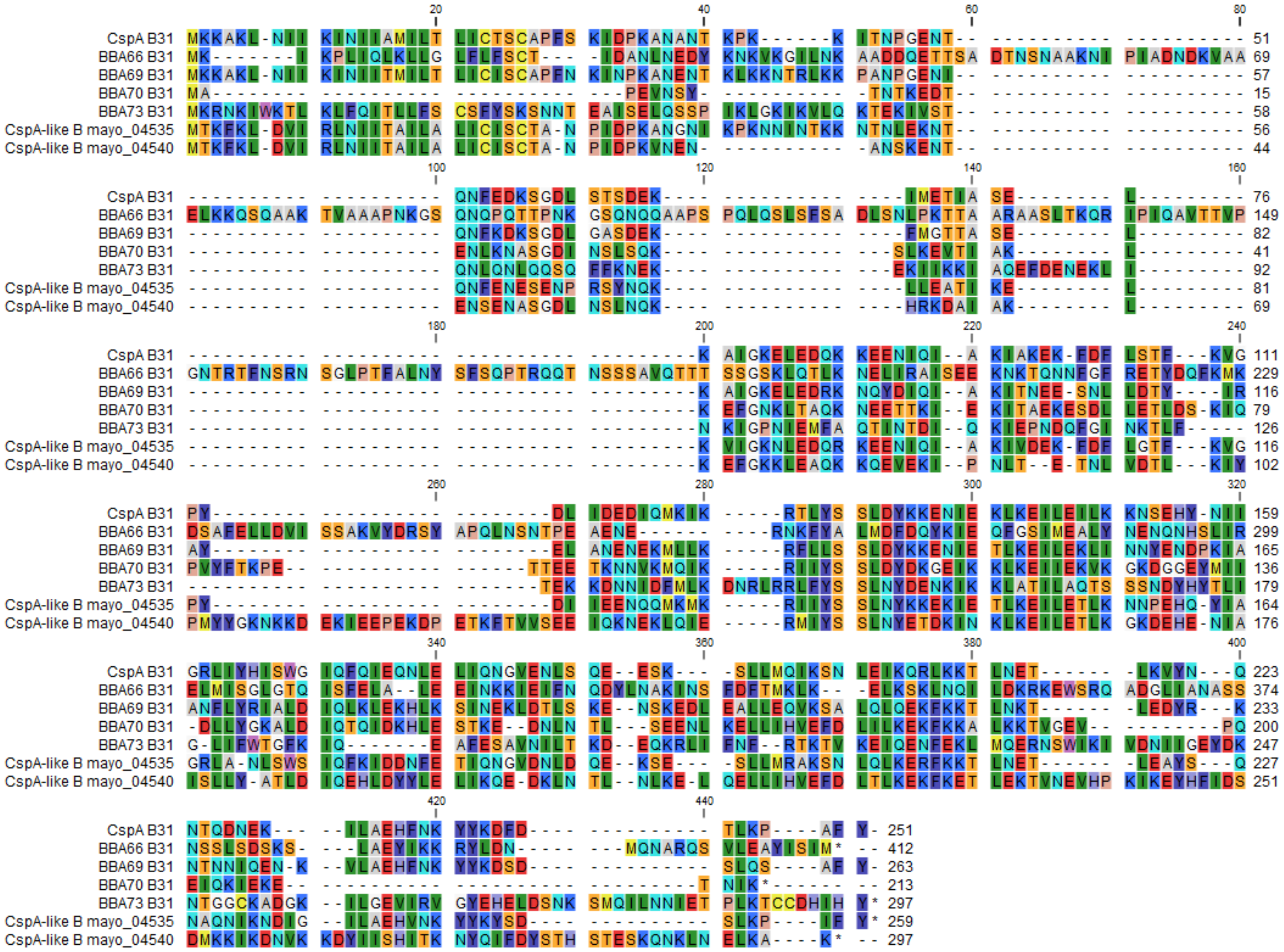

B

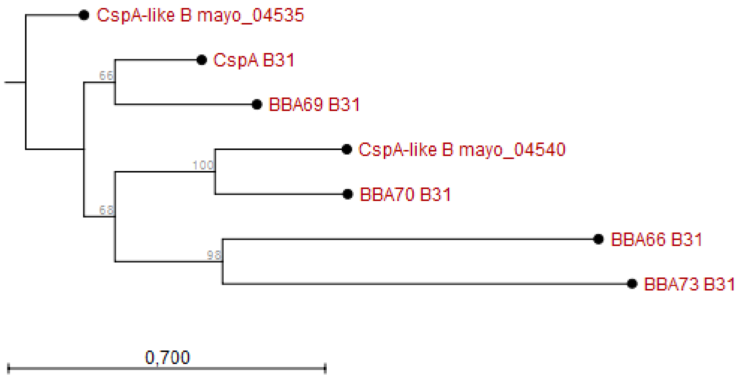

Sequence alignment and phylogenetic tree of CspA homologs of *B. burgdorferi* and CspA-like proteins of *B. mayonii*. Deduced amino acid sequences of CspA (BBA68), BBA66, BBA69, BBA70, BBA73, CspA-like Bmayo_04535, and CspA-like Bmayo_04540 were used to create an alignment and the phylogenetic tree based on the Neigbor joining method by CLC Sequence Viewer, version 8.0 (QIAGEN Aarhus A/S, Denmark).
