# Supplementary material for: Elucidating the Immune Evasion Mechanisms of Borrelia mayonii, the Causative Agent of Lyme Disease
Source: Front Immunol. 2019 Nov 26;10:2722. doi: 10.3389/fimmu.2019.02722 (PMC6902028; doi:10.3389/fimmu.2019.02722)
Supplement: Supplementary file 2 [file Data_Sheet_2.pdf]

Supplementary Figure 2

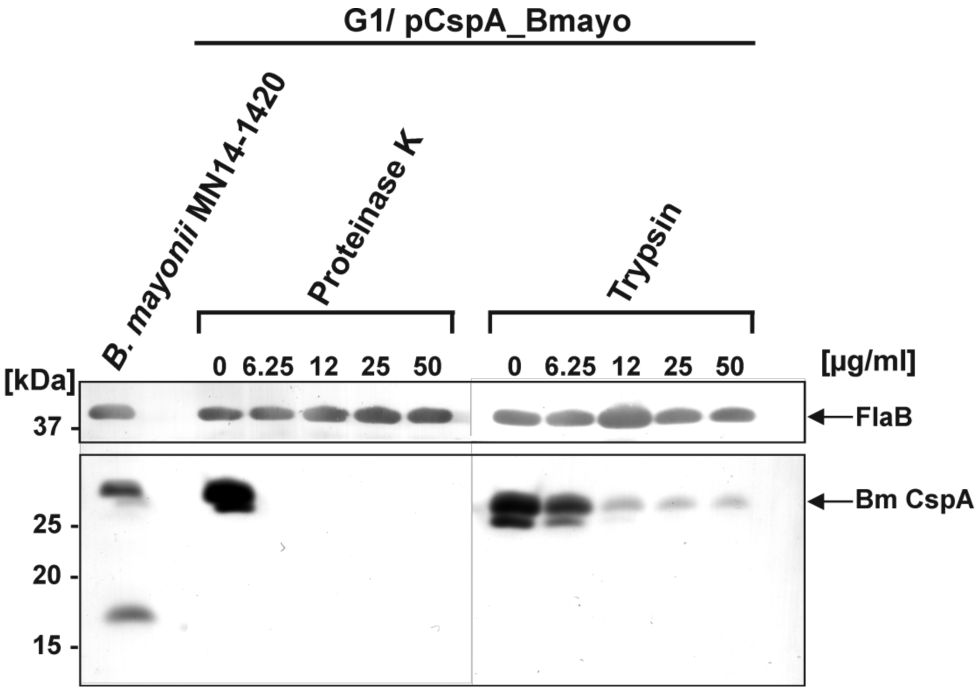

Characterization of CspA-producing spirochetes by a protease accessibility assay. An in situ protease accessibility assay was conducted to detect surface exposure of CspA of *B. mayonii* MN14-1420 on the surface of genetically-modified spirochetes. Native spirochetes were incubated with increasing concentrations of proteinase K or trypsin or without the proteases and then lysed by sonication. The cell lysates were separated by 10% Tris/tricine-SDS-PAGE and proteins were transferred to nitrocellulose membranes. Bm CspA was detected by Far Western blotting using NHS as source for FH. As a control, the periplasmatic FlaB protein was detected with the monoclonal antibody L41 1C11. The band corresponding to Bm CspA is indicated on the right. The mobilities of molecular mass standards are indicated on the left.
