# Supplementary material for: Elucidating the Immune Evasion Mechanisms of Borrelia mayonii, the Causative Agent of Lyme Disease
Source: Front Immunol. 2019 Nov 26;10:2722. doi: 10.3389/fimmu.2019.02722 (PMC6902028; doi:10.3389/fimmu.2019.02722)
Supplement: Supplementary file 3 [file Data_Sheet_3.PDF]

Supplementary Figure 3

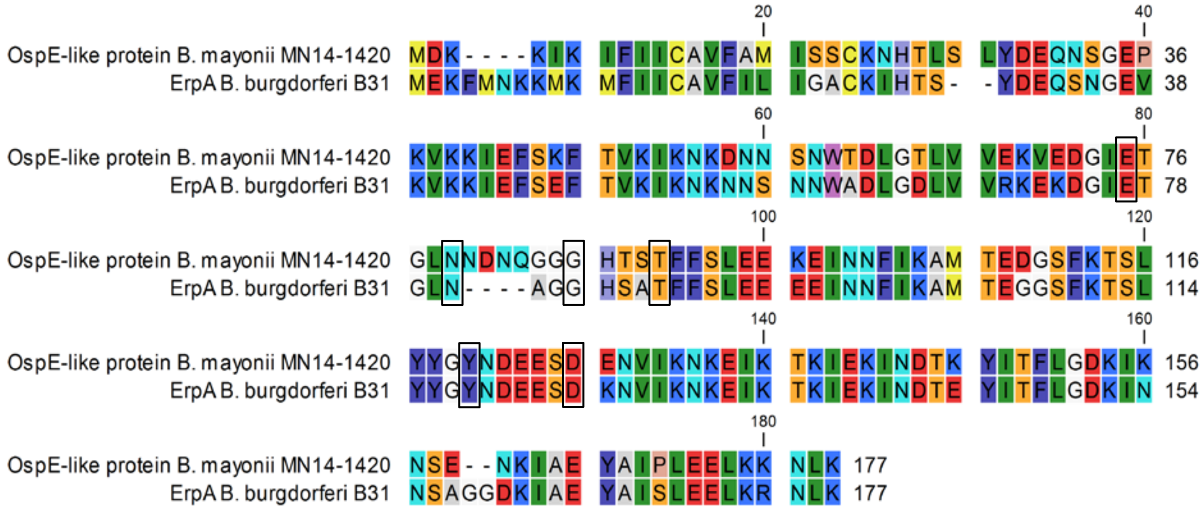

Sequence alignment of ErpA of *B. burgdorferi* B31 and the OspE-like protein of *B. mayonii*. Deduced amino acid sequences of ErpA (BBP38, NP_051199.2) and the OspE-like protein (WP_075552645.1) were used to create an alignment by CLC Sequence Viewer, version 8.0 (QIAGEN Aarhus A/S, Denmark). Residues involved in binding of ErpA to FH are boxed.
