# Supplementary material for: Elucidating the Immune Evasion Mechanisms of Borrelia mayonii, the Causative Agent of Lyme Disease
Source: Front Immunol. 2019 Nov 26;10:2722. doi: 10.3389/fimmu.2019.02722 (PMC6902028; doi:10.3389/fimmu.2019.02722)
Supplement: Supplementary file 4 [file Data_Sheet_4.pdf]

Supplementary Figure 4

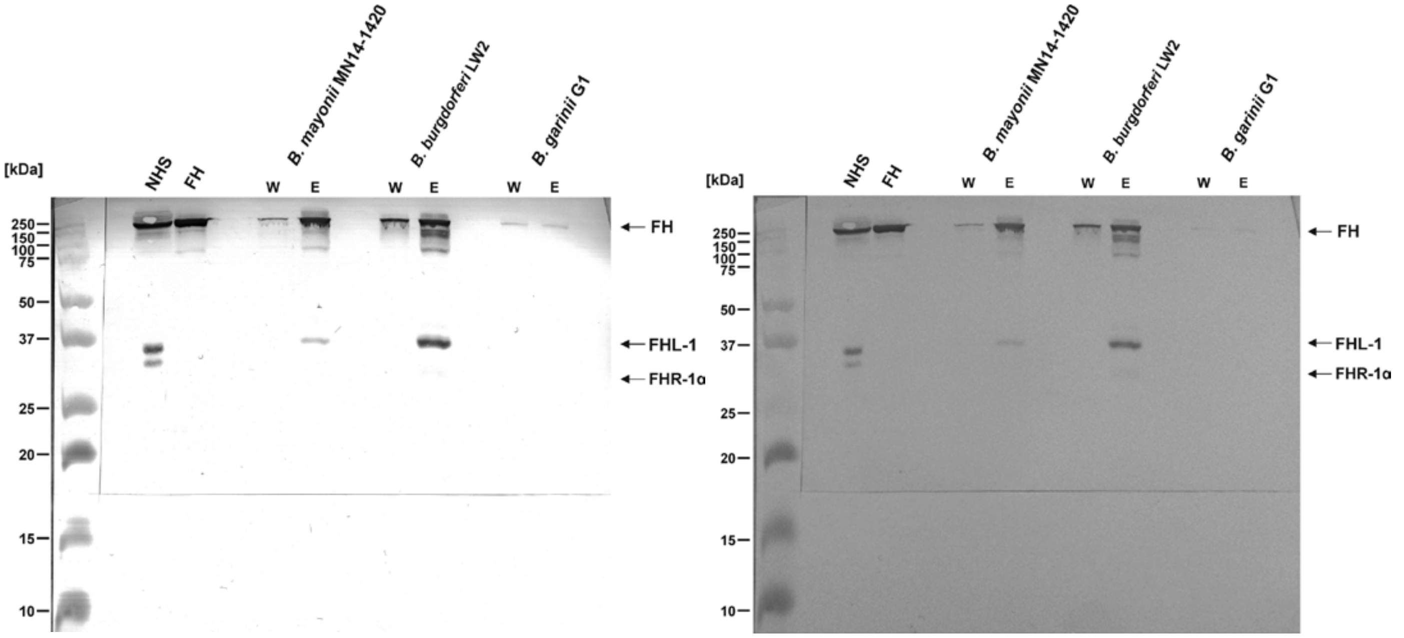

Uncropped Western blot of figure 2B. The nitrocellulose membrane was re-scanned by a GS-710 Imaging Densitometer (Bio-Rad) and for image processing the Quantity One 4.2.1 software (Bio-Rad) was used (left panel). The general settings were as follows:

- Application: Blot; HRP-Substrate (DAB)
- Filter: Green
- Resolution: 42,3 * 42,3 microns = High for small gels with tiny features

Transform operations for the re-scanned original membrane: high: 2277; low: 0; gamma: 0.71

The identical membrane was also re-scanned by a Chemidoc (Bio-Rad) and for image processing the ImageLab 4.1 software (Bio-Rad) was applied (right panel). The general settings were as follows:

- Application: Blot: Colorimetric (Filter:Standard Filter, Light: White epi illumination)

Transform operations: high 44553, low 17355, gamma 1.00

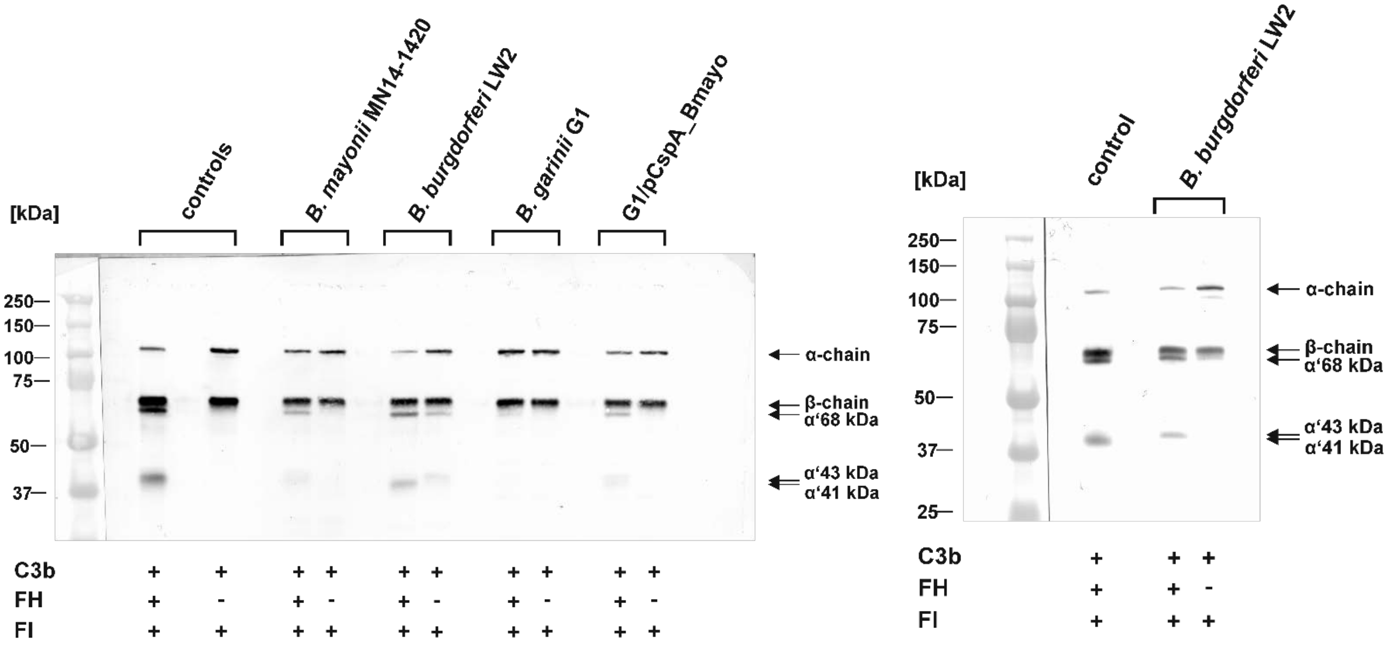

Uncropped Western blots of figure 2C. Nitrocellulose membranes were scanned by a GS-710 Imaging Densitometer (Bio-Rad) and for image processing the Quantity One 4.2.1 software (Bio-Rad) was used. The general settings were as follows:

- Application: Blot; HRP-Substrate (DAB)
- Filter: Green
- Resolution: 42,3 * 42,3 microns = High for small gels with tiny features

Transform operations of the left panel of figure 2D; 2925; low: 126; gamma: 1.09

Transform operations of the right panel of figure 2D; 3210; low: 206; gamma: 0.89
