# Supplementary material for: Elucidating the Immune Evasion Mechanisms of Borrelia mayonii, the Causative Agent of Lyme Disease
Source: Front Immunol. 2019 Nov 26;10:2722. doi: 10.3389/fimmu.2019.02722 (PMC6902028; doi:10.3389/fimmu.2019.02722)
Supplement: Supplementary file 6 [file Data_Sheet_6.PDF]

Supplementary Figure 6

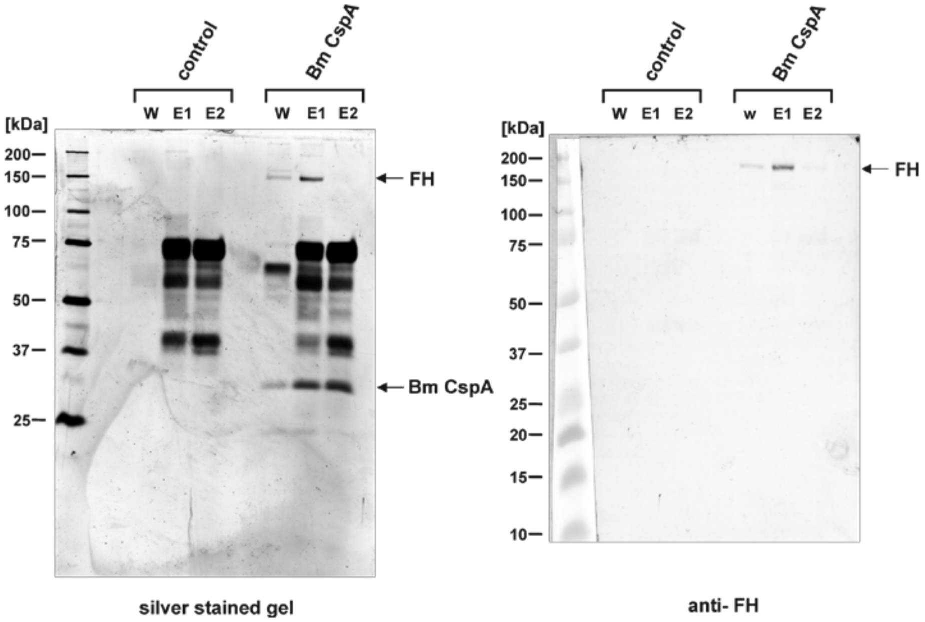

Uncropped silver-stained gel and Western blot of figure 4D. The nitrocellulose membrane was scanned by a GS-710 Imaging Densitometer (Bio-Rad) and for image processing the Quantity One 4.2.1 software (Bio-Rad) was used. The general settings were as follows:

Left panel; silver stained gel (A):

- Application: Gel
- Filter: Silver stain
- Resolution: 42,3 * 42,3 microns =High for small gels with tiny features
-

Transform operations of the silver stained gel: high: 4000; low: 1486; gamma: 4.00

Right panel, nitrocellulose membrane (B):

- Application: Blot; HRP-Substrate (DAB)
- Filter: Green
- Resolution: 42,3 * 42,3 microns =High for small gels with tiny features

Transform operations: high: 4095; low; gamma: 1.00

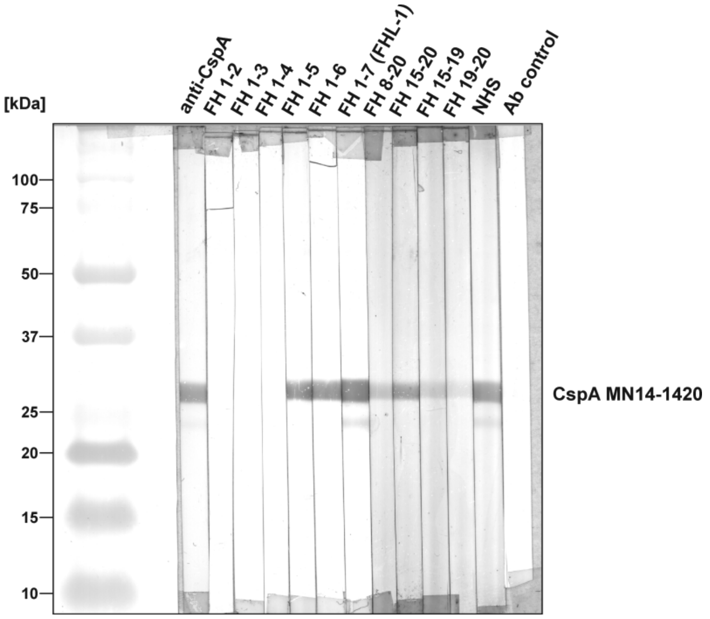

Uncropped gel and Western blot of figure 4E. Nitrocellulose membranes were scanned by a GS-710 Imaging Densitometer (Bio-Rad) and for image processing the Quantity One 4.2.1 software (Bio-Rad) was used. The general settings were as follows:

- Application: Blot; HRP-Substrate (DAB)
- Filter: Green
- Resolution: 42,3 * 42,3 microns =High for small gels with tiny features

Transform operations: high: 4095; low; gamma: 1.00
