# Supplementary material for: Elucidating the Immune Evasion Mechanisms of Borrelia mayonii, the Causative Agent of Lyme Disease
Source: Front Immunol. 2019 Nov 26;10:2722. doi: 10.3389/fimmu.2019.02722 (PMC6902028; doi:10.3389/fimmu.2019.02722)
Supplement: Supplementary file 7 [file Data_Sheet_7.PDF]

Supplementary Figure 7

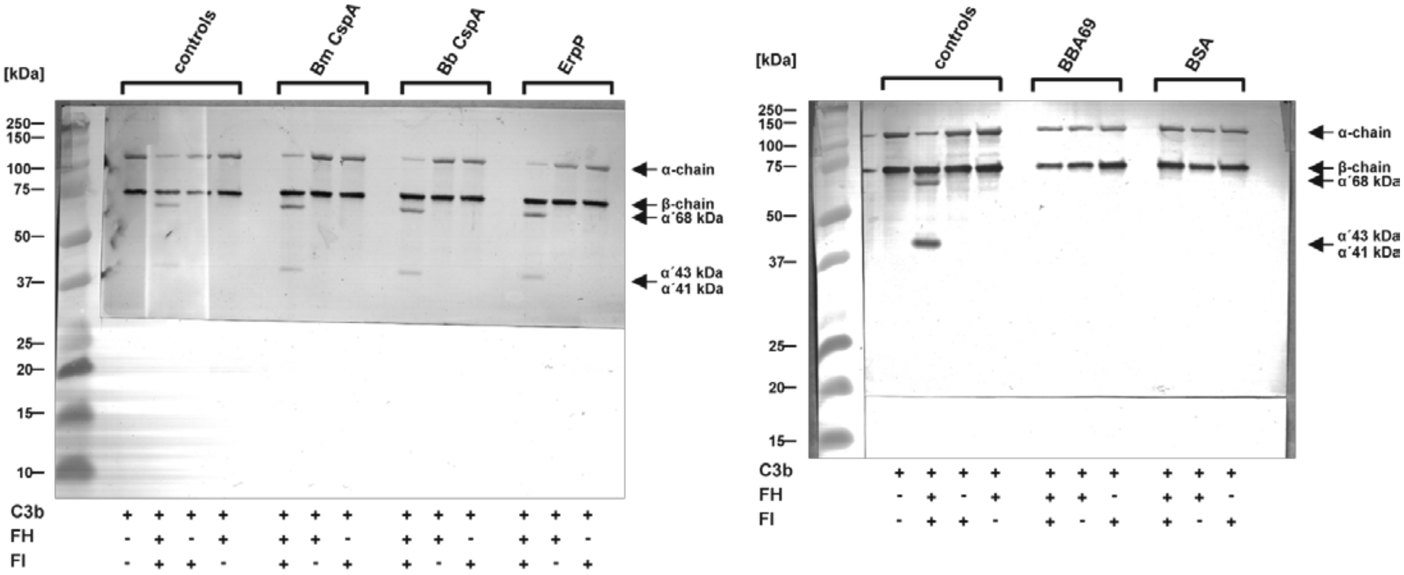

Uncropped Western blots of figure 5. Nitrocellulose membranes were scanned by a GS-710 Imaging Densitometer (Bio-Rad) and for image processing the Quantity One 4.2.1 software (Bio-Rad) was used. The general settings were as follows:

- Application: Blot; HRP-Substrate (DAB)
- Filter: Green
- Resolution: 42,3 * 42,3 microns =High for small gels with tiny features

Transform operations of the left panel: high: 2295; low: 1; gamma: 0.87

Transform operations of the right panel: high: 3826; low: 237; gamma: 1.00
