# Supplementary material for: Elucidating the Immune Evasion Mechanisms of Borrelia mayonii, the Causative Agent of Lyme Disease
Source: Front Immunol. 2019 Nov 26;10:2722. doi: 10.3389/fimmu.2019.02722 (PMC6902028; doi:10.3389/fimmu.2019.02722)
Supplement: Supplementary file 8 [file Data_Sheet_8.PDF]

Supplementary Figure 8

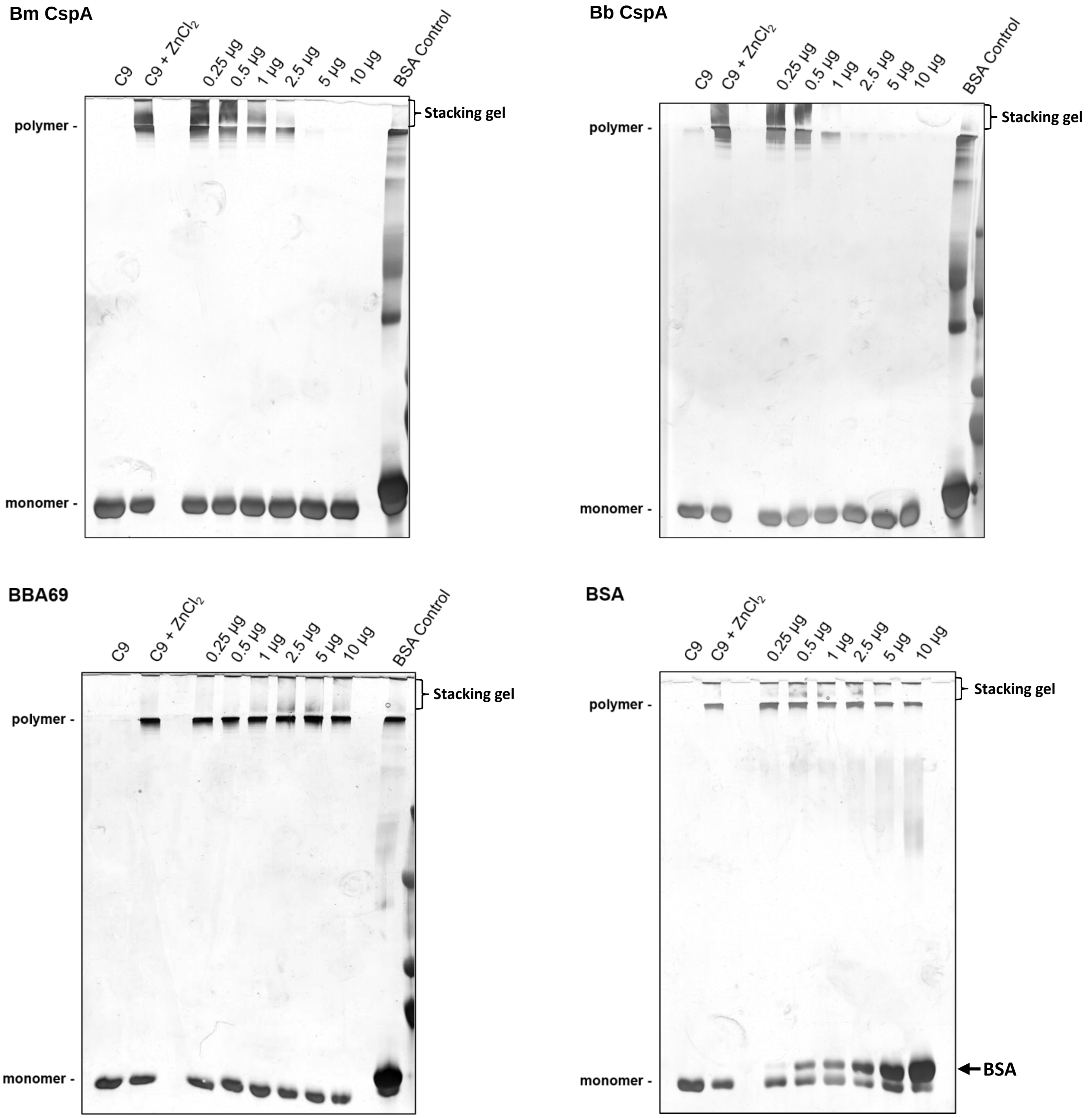

Uncropped silver-stained gels of figure 7E. SDS polyacrylamide gels stained with silver were scanned by a GS-710 Imaging Densitometer (Bio-Rad) and for image processing the Quantity One 4.2.1 software (Bio-Rad) was used. The general settings were as follows:

- Application: Gel
- Filter: Silver stain
- Resolution: 42,3 * 42,3 microns =High for small gels with tiny features
- Transform operations of the silver stained gel: high: 4095; low: 0; gamma: 1.00
