# Supplementary material for: Elucidating the Immune Evasion Mechanisms of Borrelia mayonii, the Causative Agent of Lyme Disease
Source: Front Immunol. 2019 Nov 26;10:2722. doi: 10.3389/fimmu.2019.02722 (PMC6902028; doi:10.3389/fimmu.2019.02722)
Supplement: Supplementary file 9 [file Data_Sheet_9.pdf]

Supplementary Figure 9

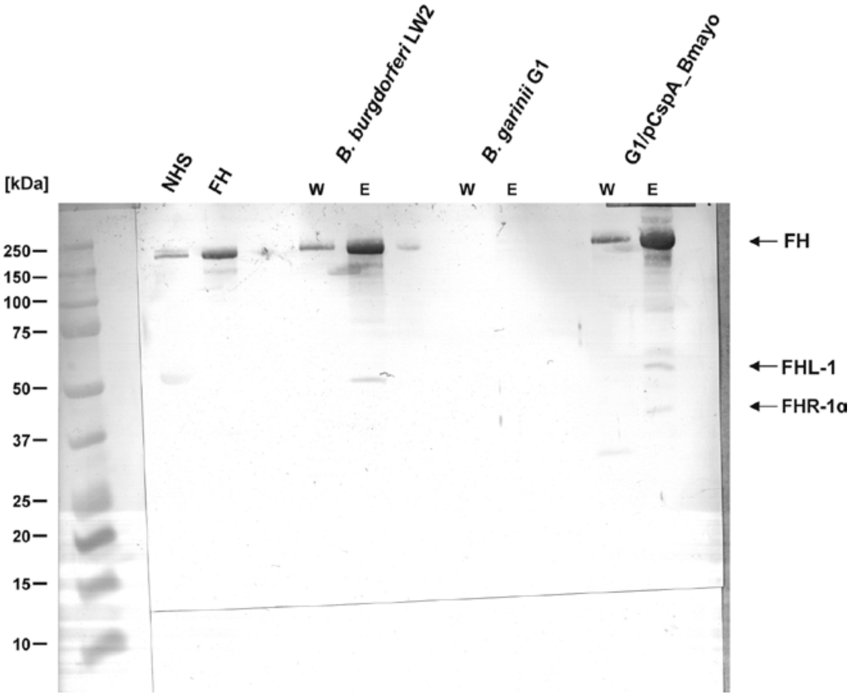

Uncropped Western blot of figure 7A. The nitrocellulose membrane was re-scanned by a GS-710 Imaging Densitometer (Bio-Rad) and for image processing the Quantity One 4.2.1 software (Bio-Rad) was used. The general settings were as follows:

- Application: Blot; HRP-Substrate (DAB)
- Filter: Green
- Resolution: 42,3 * 42,3 microns =High for small gels with tiny features

Transform operations: high: 3304; low: 47; gamma: 0.91
