# Supplementary material for: Elucidating the Immune Evasion Mechanisms of Borrelia mayonii, the Causative Agent of Lyme Disease
Source: Front Immunol. 2019 Nov 26;10:2722. doi: 10.3389/fimmu.2019.02722 (PMC6902028; doi:10.3389/fimmu.2019.02722)
Supplement: Supplementary file 10 [file Data_Sheet_10.PDF]

Supplementary table 1. Oligonucleotides used in this study

Oligonucleotide	Sequence (5'-3')[a]	Use in this work
CspA_Bmayo BamHI	GCTTTGATTTGCATTTCAGGATCCGCTAATCCAATTGATCCAAAAGCAAATG	Cloning of the *cspA* encoding gene 04535 of *B. mayonii* MN14-1420 into expression vector pQE-30 Xa
CspA_Bmayo SalI	CTCTTTTTAAAAATATCAAGGTCGACATATTATTTTTCTTGTATTAGTAAAAGATGGG	Cloning of the *cspA* encoding gene 04535 of *B. mayonii* MN14-1420 into expression vector pQE-30 Xa
CspA_Bmayo_2 BamHI	CAAAATATTATTCCTGGATCCCTTTTTAATGAA	Cloning of the *cspA* encoding gene 04535 with adjacent sequences of *B. mayonii* MN14-1420 into shuttle vector pKFSS1
CspA_Bmayo SphI	GATTACTCTACTCAGCATGCTGAATCTAAAC	Cloning of the *cspA* encoding gene 04535 with adjacent sequences of *B. mayonii* MN14-1420 into shuttle vector pKFSS1
M13 for	GTAAAACGACGCCCAGT	PCR amplification and sequencing
M13 rev	CAGGAAACAGCTATGAC	PCR amplification and sequencing
pQE-FP-30	TTGCTTTGTGAGCGGATAAC	Sequencing
pQE-RP	CTGAGGTCATTACTGGATCTATC	Sequencing

[a], Sequences of specific restriction endonuclease recognition sites are underlined
