# Supplementary material for: Elucidating the Immune Evasion Mechanisms of Borrelia mayonii, the Causative Agent of Lyme Disease
Source: Front Immunol. 2019 Nov 26;10:2722. doi: 10.3389/fimmu.2019.02722 (PMC6902028; doi:10.3389/fimmu.2019.02722)
Supplement: Supplementary file 11 [file Data_Sheet_11.pdf]

Supplementary Materials and Methods

Recombinant proteins and generation of expression and shuttle vectors

The generation of vectors producing amino-terminally hexahistidine (His_6)-tagged proteins BBA69, ErpP, and CspA of *B. burgdorferi* LW2, and BGA66 of *B. bavariensis*, respectively, are described in detailed previously (1-4).

The CspA-like encoding gene Bmayo_04535 was amplified by PCR using primers CspA_Bmayo BamHI and CspA_Bmayo SalI. Following PCR amplification and digestion with appropriate restriction endonucleases, the DNA fragment was cloned into the expression vector pQE-30 Xa (Qiagen, Hilden, Germany). The resulting plasmid pQE CspA_Bmayo was used to transform *E. coli* JM109 cells. Plasmid DNA was isolated from selected clones and sequenced to ensure that no mutations were incorporated during PCR and the cloning procedure. Production of recombinant proteins in *E. coli* was induced with isopropyl-β-D-thiogalactopyranoside. Cells were harvested by centrifugation, and sediments were lysed with a MICCRA D-9 dispersion device (Art Prozess- & Labortechnik, Müllheim, Germany) in lysis buffer containing 10 mM Imidazole, 300 mM NaCl, 50 mM NaH_2PO_4 and 1 mg/ml lysozyme (pH 8.0). Following centrifugation to clear cell debris, proteins were purified using HisPur Ni-NTA resin (ThermoFisher Scientific, Waltham, MA, USA). Fractions collected were subjected to 10% Tris/tricine SDS-PAGE followed by silver staining to assess purity of the samples. Protein concentrations were determined by bicinchoninic acid protein assay (ThermoFisher Scientific, Waltham, MA, USA).

To obtain a DNA fragment encompassing the CspA-like encoding gene Bmayo_04535 and the adjacent regulatory regions, genomic DNA isolated from *B. mayonii* strain MN14-1420 was used as a template for PCR with primers CspA_Bmayo_2 BamHI and CspA_Bmayo SphI (**see Supplementary Table 1**). Following amplification and digestion, the DNA fragment was cloned into the shuttle vector pKFSS1. Plasmids were prepared from presumptive *E. coli* clones with the Monarch plasmid kit (New England Biolabs, Frankfurt, Germany) and DNA inserts were sequenced by a commercial provider (Eurofins Genomics, Ebersberg, Germany).

SDS-PAGE, Western blot, and Far-Western blot analysis

Purified His_6-tagged proteins (500 ng each) were separated by 10% Tris/tricine SDS-PAGE and transferred to nitrocellulose membranes. The membranes were then blocked with 5% nonfat dry milk in TBS containing 0.1% Tween 20. After three wash steps with 0.1% TBS-T, membranes were incubated with a mAb L41 1C11 (anti-FlaB) (1:100) or an anti-His antibody (1:3,000) followed by horseradish peroxidase-conjugated anti-mouse immunoglobulins (1:1,000). Protein-antigen complexes were detected by tetramethylbenzidine as substrate. For the identification of FH and FHL-1 binding proteins in *Borrelia* strains and the CspA protein interacting domains in FH, Far-Western blotting was employed as previously described (5).

Enzyme-linked immunosorbent assay

Nunc MaxiSorp 96-well microtiter plates (Thermo Fisher Scientific) were coated with 100 µl of His_6-tagged proteins and BSA (each 5 µg/ml) in PBS at 4 °C overnight with gentle agitation. Following three wash steps with PBS containing 0.05% (v/v) Tween 20 (PBS-T), wells were blocked with Blocking Buffer III BSA (AppliChem, Darmstadt, Germany) for 2 h at RT. Wells were then washed three times with PBS-T and incubated with 100 µl FH or FHL-1 (5 µg/ml) at RT for 1 h. Following incubation, wells were washed thoroughly with PBS-T and incubated with a polyclonal goat anti-FH antiserum (1:1,000) for 1 h at RT. After washing three times with PBS-T, wells were incubated with HRP-conjugated anti-goat immunoglobulins (1:2,000) at RT for 1 h. The reaction was developed with *o*-phenylenediamine (Merck, Darmstadt, Germany) and the absorbance was measured at 490 nm using an ELISA reader (PowerWave HT, Bio-Tek Instruments, Winooski, VT, USA) and the Gen5 software (Bio-Tek Instruments). Additionally, to determine dose-dependency of FH binding and to calculate the dissociation constant, immobilized Bm CspA was incubated with increasing amounts of FH.

Binding of FH to spirochetes was also assessed by ELISA. Briefly, bacterial cells (2×10^7 cells) in 100 µl PBS were immobilized to Nunc MaxiSorp 96-well microtiter plates at 4 °C overnight and binding of FH was detected as described above.

Complement inactivation assays

The inhibitory capacity of CspA protein of *B. mayonii* MN14-1420 on the classical (CP), Lectin (LP), and alternative pathway (AP) was assessed by ELISA. Nunc MaxiSorp 96-well microtiter plates were coated with either human IgM (30 ng/ml) (Merck, Darmstadt, Germany) for the CP, mannan (1µg/ml) (Merck, Darmstadt, Germany) for the LP or LPS (100ng/ml) (Hycult Biotech, Beutelsbach, Germany), for the AP at 4 °C overnight with gentle agitation. Following three wash steps with TBS containing 0.05% (v/v) Triton X-100 (TBS-T), wells were blocked with PBS-T for 2 h at RT. NHS (1% for the CP, 2% for the LP, and 15% for the AP) was then pre-incubated with increasing concentrations (2.5, 5, and 10 µg) of His_6-tagged proteins for 15 min at RT before added to the respective wells to initiate complement activation. Wells were washed three times with TBS-T and incubated with a monoclonal anti-C5b-9 antibody (1:500) (Quidel, Athens, OH; USA) at RT for 1 h. Following incubation, wells were washed thoroughly with TBS-T and incubated with HRP-conjugated anti-mouse immunoglobulins (1:1,000) at RT for 1 h. The reactions were developed with *o*-phenylenediamine and the absorbance was measured at 490 nm using an ELISA reader (PowerWave HT, Bio-Tek Instruments, Winooski, VT, USA) and the Gen5 software (Bio-Tek Instruments).

Additionally, a hemolytic assay was performed to examine the inhibitory potential of the CspA protein of *B. mayonii* MN14-1420 on the terminal pathway. Briefly, NHS was pre-incubated with increasing concentrations of His_6-tagged proteins for 30 min at 37 °C. Sensitized sheep erythrocytes (1.5×10^7 cells) were pre-incubated with C5b-6 (1.5 µg/ml) for 10 min at RT. In parallel, complement C7 (2 µg/ml), C8 (0.4 µg/ml) and C9 (2 µg/ml) were pre-incubated with or without increasing concentrations (2.5, 5 or 10 µg) of His_6-tagged proteins for 5 min at RT. After 30 min at 37 °C, erythrocytes were sedimented and hemolysis was determined by measuring the absorbance of the supernatant at 414 nm.

Serum bactericidal assay

Spirochetes grown at mid-logarithmic phase were sedimented by centrifugation and resuspended in 500 µl BSK medium. Reaction mixtures consisting of 50 µl highly viable spirochetes (1×10^7), 50 µl of NHS were incubated at 37 °C with gentle agitation. The percentage of motile and viable cells was determined at different time points (1, 2, 4, and 6 h) by dark field microscopy. Nine microscopy fields were counted for each time point per analyzed strain. Each test was performed at least three times.

Serum adsorption assay

Spirochetes grown at mid-logarithmic phase were harvested by centrifugation and resuspended in 500 µl GBS (Complement Technology, Tyler, Texas). Bacteria (2×10^9) were sedimented by centrifugation and cells were then resuspended in 750 µl NHS supplemented with 34 mM EDTA (NHS-EDTA) and incubated for 1 h at RT. After three wash steps with PBSA (0.15 M NaCl, 0.03 M phosphate, 0.02% sodium azide, pH 7.2) containing 0.05% Tween-20, the proteins bound to the spirochetes were eluted by incubation with 0.1 M glycine-HCl, pH 2.0, for 15 min. After neutralization of the eluate fraction by adding 1 M Tris-HCl (pH 9.0), the bacterial cells debris were sedimented by centrifugation and soluble proteins in the supernatant were analyzed by SDS-PAGE and Western blotting as previously described (5).

C3b degradation assay

Factor I-mediated C3b inactivation in the presence of spirochetes was assayed after pre-incubation of 1×10^8 borrelial cells with PBS supplemented with 1 µg/ml FH for 1 h at RT. Thereafter, cells were washed twice with PBS and finally resuspended in 30 µl PBS containing 10 µg/ml C3b and 20 µg/ml factor I and incubated for 1 h at 37°C under gently

agitation. The bacterial cells were sedimented by centrifugation and soluble proteins in the supernatant were analyzed by SDS-PAGE and Western blotting.

Cofactor activity of protein-bound FH was analyzed by monitoring factor I-mediated conversion of C3b to iC3b using ELISA. Briefly, recombinant proteins (100 ng/ml) were immobilized on Nunc MaxiSorp 96-well microtiter plates at 4 °C overnight with gentle agitation. Following three wash steps with PBS containing 0.05% (v/v) Tween 20 (PBS-T), wells were blocked with Blocking Buffer III BSA (AppliChem, Darmstadt, Germany) for 2 h at RT. Wells were washed three times with PBS-T and incubated with FH (100 ng/ml) for 1 h at RT. After washing with PBS-T, PBS containing C3b (10 µg/ml) and factor I (20 µg/ml) was added to the wells and reactions were incubated for 1 h at 37°C. Supernatants collected from each reaction were mixed with SDS-PAGE sample buffer and loaded on a 10% Tris/tricine SDS gel. C3b inactivation products were then analyzed by Western blotting using a polyclonal anti-C3 antibody.

Determination of the inhibitory capacity of CspA of *B. mayonii* MN14-1420 on C9 polymerization

The inhibitory capacity of the CspA protein of *B. mayonii* MN14-1420 on C9 polymerization was assessed by incubating complement C9 with purified borrelial proteins. Complement component C9 (3 µg) was incubated with increasing concentrations (0.25-10 µg) of Bm Bmayo, Bb CspA, BBA69, and BSA at 37°C for 40 min. Thereafter, 50 µM $ZnCl_2$ in 20 mM Tris-HCl pH 7.2 was added to each reaction mixture for 2 h at 37°C. As further control, purified C9 was assayed under identical conditions with or without 50 µM $ZnCl_2$ to induce auto-polymerization. Reaction mixtures were then loaded on 8% Tris/Tricin-SDS gels and monomeric and polymeric C9 molecules were visualized by silver staining.

Pull-down assay for detecting interacting serum proteins

Purified His_6-tagged proteins (40 µg) were incubated with magnetic beads (Dynabeads®) coated with cobalt ions as recommend by the manufacturer (Thermo Fisher Scientific). After four washes with PBS-T (50mM phosphate, 300mM NaCl, 0.02% Tween 20), beads were incubated with 500 µl NHS-EDTA for 1 h on ice. After extensive washing with PBS-T, bound proteins were eluted with 100 mM glycine-HCl (pH 2.0) for 15 min on ice and the suspensions were neutralized by adding 1 M Tris-HCl (pH 9.0). The eluate and the last wash fraction were separated by 10% Tris/tricine SDS-PAGE under non-reducing conditions followed by staining with silver.

Transformation and characterization of serum-sensitive *B. garinii* producing CspA of *B. mayonii* MN14-1420

A high-passage, non-infectious *B. garinii* strain G1 choosen as surrogate strain was grown in 100 ml BSK medium (Bio&SELL) and electrocompetent cells were prepared as described previously (6). 50 µl aliquots of competent cells were electroporated at 12.5kV/cm with 20 µg of vector DNA. Cells were immediately diluted into 10 ml fresh BSK medium and incubated without antibiotic selection for 24 h at 33°C. Bacteria were further diluted into 90 ml BSK medium containing streptomycin (25µg/ml) and 200 µl aliquots were seeded into 96-well cell culture plates. Selected clones were expanded in 1 ml fresh BSK medium without antibiotic selection for seven days and transferred into 10 ml fresh BSK medium containing streptomycin (50 µg/ml).

Clones harboring the vector with the CspA encoding gene were sedimented by centrifugation, washed with PBS, and suspended in 50 µl of ddH_2O. Five microliters were used for PCR amplification with oligonucleotide primers M31 For and M31 Rev (**see Supplementary table 1**) at a final concentration of 100 nM each, plus 200 µM dNTPs. PCR was carried out for 25 cycles using the following parameters: denaturation at 94 °C for 60 s, annealing at 50 °C for 60 s, and extension at 72 °C for 90 s.

In situ surface assessment of CspA of *B. mayonii* MN14-1420 on spirochetes by a protease degradation assay

Surface localization of the CspA protein of *B. mayonii* MN14-1420 was assessed by an in situ protease degradation assay as previously described in detail (6). Borrelial cells were sedimented by centrifugation, washed twice, resuspended in PBS, and counted. Reaction mixtures containing 2×10^8 spirochetes and increasing concentrations of proteinase K and trypsin (6.25 to 50 µg/ml), respectively, were incubated for 2 h at room temperature. All reactions were terminated by adding 5 µl phenylmethylsulfonyl fluoride (Merck, Darmstadt, Germany) and 71 µl of cOmplete™ protease inhibitor cocktail (Merck, Darmstadt, Germany). Following sedimentation, cells were washed twice with PBS and lysed 5 times by sonication. Borrelial lysates were then subjected to Tris/tricine SDS-PAGE under reducing conditions and respective proteins were then detected by Far Western blotting (FH binding) or by Western blotting (FlaB) as described above.

References

1. C. Hammerschmidt, A. Koenigs, C. Siegel, T. Hallstrom, C. Skerka, R. Wallich, P. F. Zipfel and P. Kraiczy: Versatile roles of CspA orthologs in complement inactivation of serum-resistant Lyme disease spirochetes. *Infection and Immunity*, 82(1), 380-92 (2014).
2. C. Hammerschmidt, Y. Klevenhaus, A. Koenigs, T. Hallstrom, V. Fingerle, C. Skerka, K. M. Pos, P. F. Zipfel, R. Wallich and P. Kraiczy: BGA66 and BGA71 facilitate complement resistance of *Borrelia bavariensis* by inhibiting assembly of the membrane attack complex. *Molecular Microbiology*, 99(2), 407-24 (2016).
3. P. Kraiczy, E. Rossmann, V. Brade, M. M. Simon, C. Skerka, P. F. Zipfel and R. Wallich: Binding of human complement regulators FHL-1 and factor H to CRASP-1 orthologs of *Borrelia burgdorferi Wien Klin Wochenschr*, 118(21-22), 669-676 (2006)
4. B. Stevenson, J. L. Bono, T. G. Schwan and P. Rosa: Borrelia burgdorferi Erp proteins are immunogenic in mammals infected by tick bite, and their synthesis is inducible in cultured bacteria. Infect. Immun., 66(6), 2648-2654 (1998).
5. P. Kraiczy, C. Skerka, M. Kirschfink, V. Brade and Peter F. Zipfel: Immune evasion of Borrelia burgdorferi by acquisition of human complement regulators FHL-1/reconectin and Factor H. Eur J Immunol, 31(6), 1674-1684 (2001).
6. K. Hartmann, C. Corvey, C. Skerka, M. Kirschfink, M. Karas, V. Brade, J. C. Miller, B. Stevenson, R. Wallich, P. F. Zipfel and P. Kraiczy: Functional characterization of BbCRASP-2, a distinct outer membrane protein of Borrelia burgdorferi that binds host complement regulators factor H and FHL-1. Mol Microbiol, 61(5), 1220-1236 (2006).
